# Genetic association study of dyslexia and ADHD candidate genes in a Spanish cohort: Implications of comorbid samples

**DOI:** 10.1371/journal.pone.0206431

**Published:** 2018-10-31

**Authors:** Mirian Sánchez-Morán, Juan Andrés Hernández, Jon Andoni Duñabeitia, Adelina Estévez, Laura Bárcena, Aintzane González-Lahera, María Teresa Bajo, Luis J. Fuentes, Ana M. Aransay, Manuel Carreiras

**Affiliations:** 1 BCBL-Basque Center on Cognition Brain and Language, Donostia-San Sebastian, Gipuzkoa, Spain; 2 CIC bioGUNE, Derio, Bizkaia, Spain; 3 Universidad de La Laguna, Tenerife, Spain; 4 CIBERehd, Instituto de Salud Carlos III, Madrid, Spain; 5 Research Center for Brain, Mind & Behavior, Universidad de Granada, Granada, Spain; 6 Universidad de Murcia, Murcia, Spain; 7 Ikerbasque, Basque Foundation for Science, Bilbao, Spain; 8 Universidad del Pais Vasco UPV/EHU, Leioa, Bizkaia, Spain; University of Zaragoza, SPAIN

## Abstract

Dyslexia and attention deficit hyperactivity disorder (ADHD) are two complex neuro-behaviorally disorders that co-occur more often than expected, so that reading disability has been linked to inattention symptoms. We examined 4 SNPs located on genes previously associated to dyslexia (*KIAA0319*, *DCDC2*, *DYX1C1* and *FOXP2*) and 3 SNPs within genes related to ADHD (*COMT*, *MAOA* and *DBH*) in a cohort of Spanish children (N = 2078) that met the criteria of having one, both or none of these disorders (dyslexia and ADHD). We used a case-control approach comparing different groups of samples based on each individual diagnosis. In addition, we also performed a quantitative trait analysis with psychometric measures on the general population (N = 3357). The results indicated that the significance values for some markers change depending on the phenotypic groups compared and/or when considering pair-wise marker interactions. Furthermore, our quantitative trait study showed significant genetic associations with specific cognitive processes. These outcomes advocate the importance of establishing rigorous and homogeneous criteria for the diagnosis of cognitive disorders, as well as the relevance of considering cognitive endophenotypes.

## Introduction

Dyslexia [MIM: 127700] and Attention Deficit Hyperactivity Disorder (ADHD [MIM:143465]) are two neurobehavioral disorders with high prevalence [1]. Both are considered complex disorders influenced by multiple genetic and environmental risk factors [1,2] and it is thought that many genes are implicated in their development, each one contributing with a small effect on the total phenotypic variance. Dyslexia is characterized by difficulties in learning to read despite normal intelligence, educational opportunities or physical abilities (correct vision and audition). On the other hand, ADHD is described with symptoms such as excessive motor activity, impulsiveness and inattention [3]. ADHD is frequently divided into three subtypes depending on the predominant symptoms: the inattentive type, the hyperactive-impulsive type or the combined type [3]. In this study, we have only focused on children with inattentive symptoms (i.e. attention deficits).

Nine *loci* (DYX1-DYX9) [4] have been identified as candidates for susceptibility to dyslexia, which contain a number of genes potentially related to this disorder: KIAA0319 (6p22.3) [MIM: 609269] [5,6], *DCDC2* (6p22.1) [MIM: 605755] [6–8], *DYX1C1* (15q21.3) [MIM: 608706] [9,10], *ROBO1*(3p12)[MIM: 602430] [11], *FOXP2* (7q31) [MIM: 605317] [12]. For ADHD, molecular studies have concentrated on genes encoding proteins involved in the dopaminergic pathway, with special interest in the dopamine receptor *DRD4* (11p15.5) *[MIM*: *126452]* [13] and dopamine transporter *DAT1* (or *SLC6A3*, 5p15.3) [MIM: 126455] [14]. Other genes such as *DBH* (9q34) [MIM: 609312] [15], *COMT* (22q11.21) [MIM: 116790] [16] and *MAOA* (Xp11.3) [MIM: 309850] [17] have also been examined because of their roles in the dopaminergic system, but have not been studied extensively. Importantly, despite de fact that there have been many genetic studies of dyslexia and ADHD, conclusive results linking the two conditions/disorders is lacking.

As reported, dyslexia and ADHD appear together more often than expected [18] and some studies have shown a stronger relationship between dyslexia and symptoms of inattention [19–22] rather than hyperactivity/impulsivity symptoms. Twin and family studies suggest that this overlap is, partly, due to shared genetic background [21,23,24]. Indeed, several studies have demonstrated intersecting genetic regions for these syndromes [25,26]. The search for dyslexia or ADHD-specific susceptibility genes is difficult not only because of the complexity and heterogeneity of these conditions, but also due to their co-occurrence (also known as comorbidity) with other cognitive disorders, which makes it even more difficult to obtain conclusive results, particularly if the other disorders are not diagnosed. When comorbidity is ignored, it can be erroneously concluded that a particular variable is associated with a given disorder, when in fact it is interacting with the comorbid condition [27]. Importantly, the results of genetic analyses can only be as good as the symptomatology criteria of the studied phenotype. Therefore, it is of vital importance to correctly establish the diagnostic criteria applied to the cohorts in order to find meaningful associations [28]. In the study of dyslexia candidate genes in cases of ADHD (or the other way around, ADHD candidate genes in cases of dyslexia), these are fundamental considerations.

The aim of this study was to evaluate the reproducibility of association between reported SNPs in dyslexia and ADHD in our Spanish cohort, examining the relationship when comorbid individuals are included or excluded in the case population. In addition to each single marker approach, we have investigated the effect of composite genotypes using pairs of SNPs, to assess the epistatic relation of those markers with these cognition disorders.

Nonetheless, the diagnostic of all-or-none, when assessing whether the disorder is present or not, may not be optimal for many genetic studies, as this type of characterization does not capture the complete essence of the phenotype [1]. Both dyslexia and ADHD might arise from many different cognitive processes and, consequently, it has been suggested that identifying susceptibility genes for endophenotypes may prove a very fruitful strategy [2,29]. Many of these cognitive functions seem to be continuously distributed in the general population. Therefore, in addition to searching for genetic differences between cases and controls, we also investigated the correlation of the output genotypes and phenotypes of all participants (including the extreme ones), since direct analysis of continuous indices of severity or cognitive traits may allow discovery of genes related to the specific cognitive processes underlying each disorder [30,31].

## Materials and methods

### 1.-Sample collection and DNA extraction

A total of 4678 saliva samples were collected from children at schools all over Spain from different regions (Andalucía, Basque Country, Castilla-León, Murcia, and Canary Islands), after informed consent was obtained from their tutors or parents. La-Laguna University Ethical Committee approved consent by tutors or parents. Parents were informed and provided the opportunity to opt-out in cases in which consent was obtained from tutors. A saliva sample was collected from each participant with Oragene saliva kits (OG-500, DNA Genotek Inc., Canada) and corresponding DNA was extracted following manufacturer’s instructions, quantified and qualified on 0.8%Agarose-1xTAE-gels. Only participants with Spanish origins were used for subsequent analyses. The Spanish origin was assessed by principal component analysis (PCA) using samples from another research study diagnosed with the same criteria (a total of 1500 samples that overlapped with this study samples). A genome-wide genotyping was performed (638592 SNPs along the genome) and population stratification was analyzed by PCA. The results showed no population stratification. See S1 Fig. for further details.

### 2.-Diagnostic criteria

Dyslexics and controls were selected by using a discriminant function created with an *a priori* group diagnosed with dyslexia (n = 43) and another *a priori* group of controls taking into account their performance (one standard deviation above the mean) in a text-comprehension test (n = 470). We used a text-comprehension test to define the control group because it involves decoding and reading to avoid circularity when applying the discriminant function to word and pseudoword reading. The discriminant function successful separated the dyslexics and the controls: Wilks’ (lambda) = 0.60, F (1,497) = 329.9, p<0.0001. The variables of the discriminant function that classified dyslexics and controls with a sensibility of 91% and specificity of 94% were: age, efficiency in reading words and pseudowods, rapid naming of pictures and colors (RAN), reaction time in phoneme picture matching for phonological awareness (PA), accuracy in letter position identification, reaction time in syllable identification (see S1 File for a description of the tasks and S8 Table for mean and SD values). Table 1 shows the standardized and the structure coefficients corresponding to these variables. We selected as dyslexics the participants with an IQ (intelligence quotient) above 80 that fell in the deciles 1 or 2 of the discriminant function, and as controls the participants that fell in the decile 5 or higher.

**Table 1:** Standardized and structure coefficients for the variables that entered the discriminant function.

	Standardized	Structure
Age	-0.885	-0.025
Word and pseudoword efficiency	0.969	0.715
Rapid naming (pictures and colors)	-0.532	-0.545
Phoneme picture matching	-0.169	-0.333
Letter position identification	0.100	-0.011
Syllable identification	0.155	-0.081

Participants were classified as ADHD taking into account their error rates and reaction times in three tasks (Verbal-Stroop, Numerical-Stroop and Attentional Network Task (ANT)) (see S1 File for a description of the tasks and S9 Table for mean and SD values). Specifically, they were classified as ADHD if they had an IQ above 80 and fell in the quartile 4 on reaction times (slow responses) or in the quartile 1 in error rates (high error rates) in the three tasks. They were classified as controls when they fell in quartiles 1, 2 or 3 on reaction times. Finally, the comorbids were defined as those individuals with developmental dyslexia and ADHD (i.e. also tested and falling into both of the criteria-based categories as described above) (see S10 Table for mean and SD values).

### 3.-SNP genotyping

The DNA extracted from the collected saliva samples was characterized for seven SNP markers by Taqman SNP Genotyping assays resolved in a ViiA7 Real-Time-PCR System (Thermo-Fisher-Scientific Inc., Massachusetts, USA). The studied SNP-IDs are: rs57809907-*DYX1C1* (custom design), rs6323-*MAOA* (custom design), rs1611115-*DBH* (C_2535786_10), rs2274305-*DCDC2* (C_9344981_1_), rs4504469-*KIAA0319* (C_390135_10), rs12533005-*FOXP2* (C_220195_10) and rs4680-*COMT1* (C_25746809_50). Reactions were performed according to manufacturer’s instructions.

### 4.-Statistical analyses

**Fig 1:**
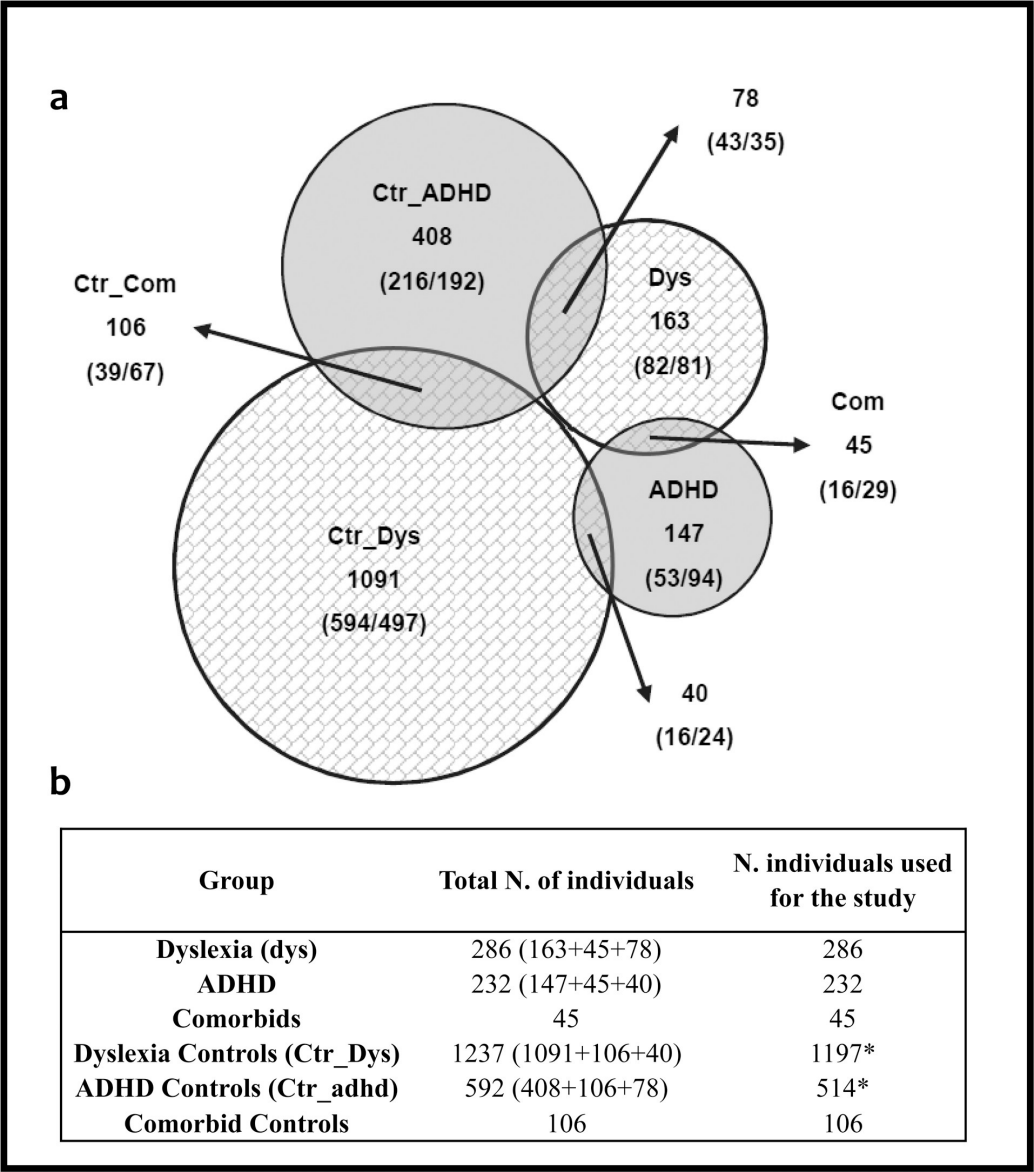
Total number of samples diagnosed for each cognitive disorder studied. a) Dys, dyslexia; ADHD, Attention Deficit Hyperactivity Disorder; Ctr__Dys_, dyslexia controls; Ctr__ADHD_, ADHD controls; Com, Comorbids; Ctr__Com_, comorbid controls. The number of females (F) and males (M) are detailed in parentheses (FFF/MMM) for each category.b) Total number of individuals in our population and number of individuals used for the study. *Note that for the Dyslexia Controls and ADHD controls we have only used samples without any disorder diagnosed. The 40 samples with ADHD have been excluded from the Ctr_Dys group and the 78 dyslexic samples have been excluded from the Ctr_adhd group for the analyses.

Resulting genotypes were tested for Hardy-Weinberg equilibrium with a χ^2^ goodness-of-fit test. Single-nucleotide-polymorphisms association analyses for genotypic, allelic, dominant and recessive models and pair-wise SNPs epistasis scrutiny in case-control strategy were carried out with PLINK (http://zzz.bwh.harvard.edu/plink/) [32] against the null hypothesis of “no association”. The samples were classified in 6 groups (Fig 1) based on their phenotype, and 7 different contrasts were performed. All the analyses were implemented in the whole cohort as well as separated by gender.

For the quantitative trait loci (QTL) approach, the genotypes of the candidate markers were analyzed for reading and attention measures by evaluating the variance with age as covariate (ANCOVA) and Hochberg false discovery rate correction [33]. Children with IQ lower than 80 were excluded from the analyses. Related to reading and attention, the variables examined were word reading (high and low frequency words), pseudoword reading, PA, RAN, syllable discrimination, Verbal-Stroop, Numerical-Stroop, and ANT (S1 File).

## Results

### Parameters of the population studied

A total of 4678 saliva samples were collected from children at schools all over Spain. Out of these, 3357 samples (1664 females, 1693 males) with ages between 6–16 years fulfilled the established criteria and, therefore, were used in the present study. Fig 1 shows the distribution of individuals diagnosed with dyslexia (Dys), ADHD or characterized as controls (Ctr__Dys_, Dyslexia Controls; Ctr__ADHD_, ADHD controls). Note that each sample could meet the criteria for more than one phenotype. Particularly, we considered as comorbids (Com) 45 participants who were classified both as dyslexic and ADHD, and we identified 106 comorbid-controls (Ctr__Com_) who shared the criteria for controls of both disorders.

### Different marker associations found depending on the inclusion/exclusion of the comorbid samples within the groups compared

SNP genotyping raw data for each comparison can be downloaded from the S1 Dataset as *.ped and *.map files. The frequencies of the analyzed SNPs in our population and in the general European population can be found in S4, S5, S6 and S7 Tables and single marker allelic associations are summarized in Table 2. Note that most of the described associations do not pass the multiple testing correction. It is worth mentioning that the statistical significance varied notably when comorbids were included or not in the case group. Interestingly, markers within *KIAA0319* and *FOXP2* were significantly related to dyslexia when comorbid samples were included (Dys+Com-*vs*-Ctr__Dys_), as well as when comorbids were analyzed as an independent group (Com-*vs*-Ctr__Dys_/Com-*vs*-Ctr__ADHD_/Com-*vs*-Ctr__Com_).

**Table 2:** Genetic association results (p values) for single-markers (allelic model).

					Dys	ADHD	Com	Com	Com	Dys+Com	ADHD+Com	
Chr	SNP	Gen	A1	A2	Ctr__Dys_	Ctr__ADHD_	Ctr__Dys_	Ctr__ADHD_	Ctr__Com_	Ctr__Dys_	Ctr__ADHD_	
6	rs4504469	*KIAA0319*	T	C	0.0913	0.5377	0.0025	0.0031	0.0101^b^	0.0084^b^	0.0952	
7	rs12533005	*FOXP2*	C	G	0.1277	0.6375	0.0079^b^	0.0015	0.0096^b^	0.0191^b^	0.1058	
6	rs2274305	*DCDC2*	T	C	0.9424	0.5819	0.2011	0.3516	0.3228	0.5935	0.8998	
15	rs57809907	*DYX1C1*	A	C	0.9353^a^	0.9315	0.3662^a^	0.1902	0.1516	0.6887^a^	0.6654	
9	rs1611115	*DBH*	T	C	0.5746	0.1664	0.8282	0.9194	0.1763	0.5555	0.2140	
22	rs4680	*COMT*	A	G	0.4648	0.5494	0.0732	0.0887	0.1361	0.9883	0.2416	
X	rs6323	*MAOA*	G	T	0.6392	0.1607	0.3279	0.5030	0.5486	0.4623	0.1362	
					241	187	45	45	45	286	232	N° Cas
					1197	514	1197	514	106	1197	514	N° Ctr

Abbreviations: Chr = chromosome, A1 = allele 1, A2 = allele 2. The grey square shows the case groups in the superior line and the control groups in the inferior one. Dys = dyslexia samples, ADHD = Attention Deficit Hyperactivity Disorder samples, Com = Comorbid samples, Ctr__Dys_ = dyslexia controls, Ctr__ADHD_ = ADHD controls, Ctr__Com_ = Comorbid controls, N° Cas = number of case samples, N° Ctr = number of control samples. Significance values <0.05 are represented underlined. a = not in Hardy-Weinberg equilibrium. b = not significant after Bonferroni correction (adjusted significance value: p<0.007).

Further associations were identified when considering paired-SNPs as an epistatic variant (Table 3). The composite genotype of *DCDC2*-*DYX1C1* SNPs presented significant association with ADHD (ADHD-*vs*-Ctr__ADHD_), with lower p-values when comorbid samples were considered jointly (ADHD+Com-*vs*-Ctr__ADHD_), and also in the Com-*vs*-Ctr__ADHD_ test. In contrast, the combination *DCDC2*-*KIAA0319* correlated with dyslexia independently of the inclusion/exclusion of comorbid samples. Additionally, rs4680-*COMT* showed significant association in the genotypic comparatives Dys+Com-*vs*-Ctr__Dys_ and Com-*vs*-Ctr__Dys_ (p-values = 0.019/0.018, respectively, S1 Table). However, when filtering these data by gender (S2 and S3 Tables), rs4680 does not appear significantly linked, although for males (S3 Table), some trend of association (p<0.1) was observed at allelic, dominant and recessive models.

**Table 3:** Genetic association results (p values) for pair-wise SNPs interactions (allelic model).

						Dys	ADHD	Com	Com	Com	Dys+Com	ADHD+Com	
Chr1	SNP2	Gene_1	Chr2	SNP2	Gene_2	Ctr__Dys_	Ctr__ADHD_	Ctr__Dys_	Ctr__ADHD_	Ctr__Com_	Ctr__Dys_	Ctr__ADHD_	
6	rs2274305	*DCDC2*	6	rs4504469	*KIAA0319*	0.0026	0.1472	0.4991	0.5163	0.4128	0.0014	0.0858	
6	rs2274305	*DCDC2*	7	rs12533005	*FOXP2*	0.2922	0.9086	0.9435	0.9228	0.9427	0.2685	0.7790	
6	rs2274305	*DCDC2*	9	rs1611115	*DBH*	0.0812	0.8711	0.1487	0.0537	0.0702	0.2807	0.3854	
6	rs2274305	*DCDC2*	15	rs57809907	*DYX1C1*	0.6073	0.0215^b^	0.0151^a^	0.0237^b^	0.0368^b^	0.6186	0.0056^b^	
6	rs2274305	*DCDC2*	22	rs4680	*COMT*	0.6632	0.3100	0.5400	0.9705	0.9121	0.4677	0.3962	
6	rs4504469	*KIAA0319*	7	rs12533005	*FOXP2*	0.2089	0.5621	0.9529	0.8237	0.3016	0.1639	0.8171	
6	rs4504469	*KIAA0319*	9	rs1611115	*DBH*	0.8744	0.6283	0.7593	0.8639	0.8407	0.9596	0.7139	
6	rs4504469	*KIAA0319*	15	rs57809907	*DYX1C1*	0.1337	0.6962	0.7166	0.8852	0.7612	0.1216	0.9075	
6	rs4504469	*KIAA0319*	22	rs4680	*COMT*	0.4945	0.6205	0.4747	0.6701	0.3619	0.5495	0.6147	
7	rs12533005	*FOXP2*	9	rs1611115	*DBH*	0.4117	0.6482	0.8208	0.4449	0.9416	0.5087	0.5522	
7	rs12533005	*FOXP2*	15	rs57809907	*DYX1C1*	0.3367	0.7178	0.9568	0.9686	0.3636	0.3526	0.6895	
7	rs12533005	*FOXP2*	22	rs4680	*COMT*	0.8976	0.5683	0.8492	0.6639	0.3706	0.9023	0.8783	
9	rs1611115	*DBH*	15	rs57809907	*DYX1C1*	0.5203	0.5009	0.1110	0.1546	0.9451	0.2098	0.2192	
9	rs1611115	*DBH*	22	rs4680	*COMT*	0.2135	0.1642	0.5489	0.8396	0.9118	0.3566	0.1835	
15	rs57809907	*DYX1C1*	22	rs4680	*COMT*	0.1337	0.9971	0.1902	0.1768	0.4815	0.0782*	0.6366	
						241	187	45	45	45	286	232	N° Cas
						1197	514	1197	514	106	1197	514	N° Ctr

Abbreviations: Chr1 = Chromosome in which is localized SNP1, Gene_1 = gene in which is localized the SNP1, Chr2 = Chromosome in which is localized SNP2, Gene_2 = gene in which is localized the SNP2. The grey square shows the compared cases group in the superior line and the control group in the inferior one. Dys = dyslexia samples, ADHD = Attention Deficit Hyperactivity Disorder samples, Com = Comorbid samples, Ctr__Dys_ = dyslexia controls, Ctr__ADHD_ = ADHD controls, Ctr__Com_ = Comorbid controls. N° Cas = number of case samples, N° Ctr = number of control samples. Significance values <0.05 are represented underlined. a = not in Hardy-Weinberg equilibrium. b = not significant after Bonferroni correction (adjusted significance value: p<0,003).

For the analysis of rs6323 located in *MAOA* at chromosome X, only female samples were considered. The results showed that rs6323 was significantly associated at the recessive comparative ADHD+Com-*vs*-Ctr__ADHD_ (p-value = 0.022, S1 Table). The recessive tests within the comorbid comparatives were not performed for rs6323 because the sample size of the compared groups was too small.

### Comorbids exhibited extreme discriminant values compared to dyslexic and ADHD samples

**Fig 2:**
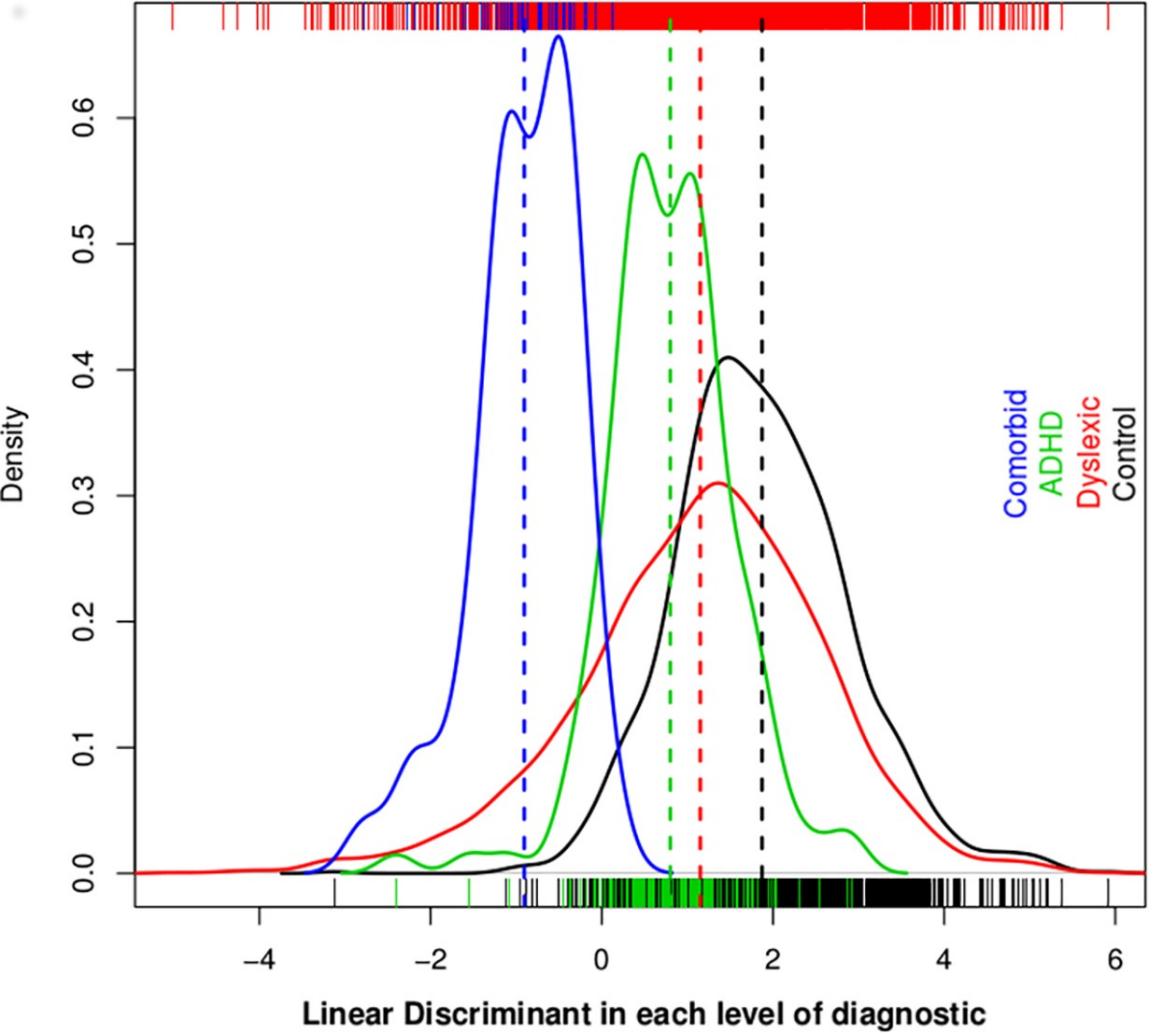
Distribution of the values of the discriminant function for each phenotypic group. Each histogram was assessed through a kernel density estimation. The comorbid samples were considered as a single group, and therefore, excluded from the dyslexia and ADHD cohorts. The vertical color bars of the upper and lower borders represent the samples of each group (blue = comorbids, green = ADHD samples, red = dyslexics and black = controls). Dashed vertical lines represent the average values of each distribution (blue = comorbids, green = ADHD samples, red = dyslexics and black = controls).

In order to understand the effect of comorbids in the analyses performed, we plotted the discriminant function values to the studied samples, which resulted in the distribution of phenotypes shown in Fig 2. According to this scattering, the comorbid group showed extreme discriminant values. The samples situated at the opposite side of the graph correspond to the controls, while the dyslexics and ADHD fall in between.

### Dyslexia and ADHD candidate SNPs are associated with cognitive traits in the general Spanish population

For QTL analyses, the genotypes and values of variables from the total population (N = 3357) were analyzed (see Table 4). Remarkably, some SNPs showed associations with tasks related either to dyslexia or to ADHD, while others showed associations with tasks related to both disorders. The four SNPs previously related to dyslexia showed very different associations. rs2274305-*DCDC2* was coherently associated with PA and RAN. Similarly, *DYX1C1* was also accordingly associated with word-reading and RAN, but also with the performance in the Verbal and Numerical-Stroop tasks that involve attentional processes. Interestingly, rs12533005-*FOXP2*, described in dyslexia studies, was related to the performance in the Verbal and Numerical-Stroop tasks. Finally, rs4504469-*KIAA0319* was related to word reading, RAN, syllable discrimination and Numerical Stroop.

**Table 4:** Genetic association of the analyzed SNPs to the variables measuring cognitive processes related to reading and attention.

Gene		Reading			PA	RAN	Syllable discrimination	STROOP		ANT
		Word-Reading		Pseudoword						
		High	Low					Verbal	Numerical	
***KIAA0319* (rs4504469)**	Omnibus	F(1,745) = 115.89 ***				F(1,753) = 16.58 ***	F(1,785) = 35.19***		F(1,792) = 8.34***	
	CT vs CC					t(753) = 2.36 *	t(785) = 2.24*		t(792) = -2.94 **	
	TT vs CC	t(745) = -2.06 *								
***FOXP2* (rs12533005)**	Omnibus							F(1,698) = 178.36 ***		
	CC vs GG							t(698) = -20.903 *	t(791) = -24.706 *	
	CG vs GG							t(698) = -23.170 *		
***DCDC2* (rs2274305)**	Omnibus				F(1,771) = 464.31 ***	F(1,756) = 16.61 ***				
	CT.vs.CC				t(771) = 22.42 *					
	TT.vs.CC					t(756) = -21.24 *				
***DYX1C1* (rs57809907)**	Omnibus	F(1,742) = 119.985 ***				F(1,750) = 16.4545 ***		F(1,697) = 48.989 ***	F(1,789) = 7.534 **	
	AA vs CC					t(750) = -20.274 *		t(697) = 21.343 *	t(789) = -32.645 **	
	AC vs CC	t(742) = -20,331 *				t(750) = -21.956 *				
***DBH* (rs1611115)**	Omnibus				F(1,763) = 457.366 ***		F(1,779) = 33.803 ***			
	TT vs CC									
	CT vs CC				t(763) = 20.274 *		t(779) = -19.907 *			
***COMT1* (rs4680)**	Omnibus									F(1,641) = 54.268 ***
	AG vs GG									t(641) = -20.913 *
	AA vs GG									
***MAOA* (rs6323)**	Omnibus	F(1,731) = 403.101 ***	F(1,729) = 402.98 ***	F(1,739) = 435.37 ***	F(1,592) = 141.128 ***	F(1,757) = 16.68 ***	F(1,771) = 273.8 ***		F(1,795) = 8.034 **	
	GT vs GG	t(731) = -2.2675 *	t(729) = -2.3324 *	t(739) = -2.2024 *	t(592) = -2.13 *		t(771) = -2.796 **			
	TT vs GG					t(757) = 2.77 **			t(795) = -2.7882 **	

High/Low = high or low frequency words, PA = phonological awareness, RAN = Rapid automatic naming, ANT = Attentional Network Test. Significant values (after Hochberg false discovery rate correction) of cognitive task to some genetic variable are displayed. Omnibus values refer to F(df1,df2) = F-value, and genotypes contrasts are shown as t(df) = t-value. Associations are specified as:

*** p<0.001

** p<0.01

* p<0.05, being p the Pvalue obtained after Hochberg correction [33].

The analyses of the three SNPs previously related to ADHD also showed very different outcomes. While rs4680-*COMT1* was associated with the ANT tasks, the rs6323-*MAOA*, a marker for ADHD, showed association not only with Numerical-Stroop but also with word and pseudoword reading time, PA, RAN and syllable discrimination. Finally, rs1611115-*DBH*, another marker for ADHD, was related to PA and syllable discrimination.

## Discussion

The present reading/attentional candidate gene association study based on case-control status and QTL approach shows that variants of selected genes are related to the phenotype and highlights the importance of accurate characterization of phenotypes. In addition, the study shows genetic associations to cognition by analyzing the relationship of specific SNPs with cognitive quantitative measures such as endophenotypes in a large sample of individuals.

In particular, we replicated previous associations for *KIAA0319* and *FOXP2* variants in dyslexic samples, with the relevant finding that higher significance values were obtained for those SNPs when comorbid samples were included. This observation is concordant with the fact that individuals who suffer from both disorders present a more severe manifestation of the symptoms as shown in previous studies [20,34] and in Fig 2. According to Wilcutt *et al*.[20] the dyslexia-ADHD comorbid condition shows a more extensive and severe profile of neuropsychological weakness. Furthermore, the heritability of reading disabilities was significantly higher in dyslexics if the individuals also met criteria for ADHD according to Willcutt *et al*.[20], which could support our data of a more significant p value when comorbids are considered. Importantly, Scerri *et al*. [7] signaled the importance of not excluding comorbid samples in association analyses, based on the statistical significance that was observed for *DCDC2* markers in dyslexic individuals, but only when comorbid samples were considered.

Interestingly, *DYX1C1*and *DCDC2* SNPs, both candidates for the susceptibility of developing dyslexia, did not show any individual association with this disorder. The lack of relationship with *DYX1C1* is not unexpected, since the significant results found in a Finnish population study of Taipale *et al*. [9] in have not been replicated in subsequent reports [10,35]. Either different causal variants or singular linkage disequilibrium patterns may exist within this gene in each population [36], or its effect may only be present under environmental disadvantage [37]. In addition, the genetic relation of the *DCDC2* variants within the dyslexia phenotype has been found to differ across the subgroup classification of the disorder [38]. Notably, we found significant effects when considering the composite genotype of SNPs within *KIAA0319* and *DCDC2* (Table 3), which have been previously reported [6,39–42]. Although these two genes are localized in the same chromosome, the lack of linkage disequilibrium between them in the studied cohort points to an independent but cooperative genetic association of these genes with dyslexia. Furthermore, the combined effect of these two genes had been described previously by Powers *et al*.’s [43], who showed that a *DCDC2* risk haplotype interacts synergistically with a *KIAA0319* haplotype, giving more extreme results when both risk haplotypes where presented together rather than separately. Also, *DCDC2* seems to interact with *DYX1C1* in our ADHD samples when both genes are analyzed epistatically. Markers previously associated to dyslexia have been found to be also linked to hyperactivity and/or inattention, as shown in Couto *el al*. [25] and Mascheretti *et a*l. [41]. *DYX1C1* was also evaluated in ADHD samples [44,45] and a haplotype of 6 SNPs was connected to the attentional symptoms of this disorder.

Concerning the previously reported allelic association of ADHD candidate SNPs in *DBH*, *COMT* and *MAOA*, none of these was replicated in our ADHD samples, although we found some significant effects following other approaches (S1 Table). *COMT* appears to be associated in the genotypic analysis to our dyslexia samples and has been connected recently to reading skill tasks (PA and spelling) [46], as well as to reading comprehension [47], adding importance to the possible role of this gene in reading-related cognitive process. As shown by Grigorenko *et al*. [47], there was significant divergence in the frequencies of 4 *COMT* haplotypes between individuals with and without comprehension difficulties.

Dyslexia and ADHD prevalence dissimilarities between males and females is well reported in numerous studies [27], and it is known that dopaminergic neurotransmission, implicated in many cognitive functions, could be modulated by gender [48]. Accordingly, the *COMT* gene has been identified in several ADHD studies as having sex specific effects [49,50] and its susceptibility alleles may differ [51]. Given this fact, *COMT* emerges as a good candidate to scrutinize for possible divergences in cognitive processes between boys and girls. Our results do not show significant differences for this *locus*, but a trend (p<0.1) is observed in our dyslexic group (S2 and S3 Tables).

An essential point that should be taken into consideration when trying to replicate results of genetic association studies is the diagnostic criteria of the samples studied, which is particularly hard to establish for certain syndromes. In many studies, dyslexia and ADHD are classified as separate clinical groups, and usually considered independently, although usually there is a substantial percentage of cases that share both conditions [19,52,53]. Despite the difficulty in defining the boundaries of certain neurological disorders and the complications of searching for genetic markers for complex traits, several *loci* have been proposed as potential biomarkers for dyslexia [4] and ADHD [54]. However, the results obtained in different studies are often contradictory and their reproducibility is limited. The heterogeneity of the criteria or the psychometric tests used to describe the categorical groups makes the comparison between studies difficult, and this might be one reason for the inconsistencies found among different studies and populations. Moreover, the ethnicity of the cohorts may also influence this low reproducibility, as the frequencies of the polymorphisms analyzed are heterogeneous around the world. Furthermore, different genetic variants for the same genes are considered in each study, and causative gene variants have been shown to be different depending on the population [55].

Given that some of the previous studies relating dyslexia or ADHD candidate genes with these disorders have shown quite inconsistent results, then perhaps establishing genetic relationships by focusing on cognitive skills, rather than centering on contrasting strictly-diagnosed dyslexia or ADHD, may produce successful results. Following this approach, our study shows some systematic relationships between cognitive quantitative traits and genes:

1. Different research groups [5,31,56–58] have found correlations of *DCDC2*, *KIAA0319* and *DYX1C1* with irregular, regular or pseudoword reading, while others did not [8,25]. The association of *KIAA0319* and *DYX1C1* with word reading was replicated in our population. In addition, we also found a relationship between reading and *MAOA*, although results obtained for this gene should be considered with caution as it is located in chromosome X and we analyzed boys and girls together.
2. Another cognitive trait directly connected to the reading process is fluency. The association of *DCDC2* with this trait has been reported previously [7,59]. A marker of *DYX1C1*, as well as several SNPs in *KIAA0319* have also been associated to the digit-RAN task [57,60,61]; the results of the current study add to this list rs4504469-*KIAA0319* and rs6323-*MAOA*, as related to the RAN.
3. Phonological decoding, which plays a central role in both normal and abnormal reading development, has been consistently reported as related to reading ability [62–64]. In fact, deficits in phonological awareness are considered the main proximal cause of cases of reading disability [65]. Dyslexia candidate genes have been correlated with tasks measuring phonological abilities, such as *DYX1C1* and *FOXP2* with short-term memory [56,58] or phonological memory [12,60], or *DCDC2* [66] and *KIAA0319* [5,7,61,66] with phoneme awareness itself. In our study, in addition to replicating the association of *DCDC2* with a phonological awareness task (reaction-time in the phoneme-picture matching task), we have also detected a possible implication of both *DBH* and *MAOA* in phonological awareness.
4. Speech perception deficits in dyslexic samples have long been proposed [67], particularly deficits in the pre-attentive and automatic information processing measured by the Mismatch Negativity (MMN) component. In fact, the MMN, established as an objective measure of speech discrimination, has been suggested as a neurophysiological endophenotype for dyslexia [68]. Furthermore, some research groups have linked MMN with genetic variants, such as *SLC2A3* [69] or rare variants in a region between the genes *KIAA0319* and *DCDC2* [70]. In the present study, we found a relationship between *KIAA0319*, *DBH* and *MAOA* with the task measuring syllable discrimination. Although they measure different processes (e.g., automatic versus attentive processing of speech), both reveal gene-cognition association in speech processing.
5. Weaknesses in executive domains such as verbal working memory, planning, and response inhibition are consistent cognitive traits in ADHD symptoms [71–73]. We failed to replicate the association found by Fosella *et al*. [74] between *MAOA* and executive attention and alerting, but the link between rs4680-*COMT* and the conflict index, based on ANT evaluation, shows that *COMT* is related to executive control. Several studies have recently associated variants of *COMT* with cognitive domains such as working memory [75] or cognitive flexibility [76]. In addition, this gene has previously been found to be associated with Stroop attentional tasks [77]. These attentional tasks are not usually analyzed with dyslexia candidate genes, therefore, one intriguing result obtained from our cohort is the relationship of this type of task with *KIAA0319*, *FOXP2* and *DYX1C1* SNPs.

Interestingly, *FOXP2* and *COMT* have been found to be associated with dyslexia and with attentional tasks in our case-control study, although these genes are not related to reading variables in the general population. *KIAA0319*, also associated with dyslexia, is associated with syllable discrimination, RAN and high frequency word reading but also with Numerical-Stroop. In addition, *DBH* and *MAOA* did not show any relationship with the defined phenotypes, but appear to have some correlation with both attention and reading-skills in the general population. This fact reinforces the importance of studying the quantitative measures used to define each phenotype considered, in order to discover evidence of cause-consequence pairs between genes and cognitive processes respectively, instead on focusing just on the categorical groups extrapolated from these quantitative measures.

We are aware of the limitations of this study and, therefore would like to specify them before concluding: (i) the hyperactivity or other possible disorder comorbidities were not diagnosed, (ii) the potential effects of the environment were not taken into account; (iii) the comorbid group is small, and (iv) the sample size in some of the comparisons performed is different.

In summary, significant association of some candidate SNPs with dyslexia and ADHD has been replicated in our Spanish population, but the significance depends on the particular phenotypic groups compared. These outcomes support the importance of a clear definition of the phenotype, especially when comorbid samples are present. Dyslexia and ADHD are complex disorders and so the search for interacting patterns of genes as well as environmental influences will give rise to more successful and reproducible results. Moreover, identification of the relationships between some DNA variants and cognitive tasks, especially when the polymorphisms themselves are not associated with any disorder, adds value to the research on endophenotypes, instead of the traditional dichotomist classification. These improvements will help us find precise and more specific genetic causes of these cognitive dysfunctions.

## Supporting information

S1 Table Association results for single markers at different genetic models. (DOCX)

S2 Table Association results for single markers at different genetic models in female samples. (DOCX)

S3 Table Association results for single markers at different genetic models in male samples. (DOCX)

S4 Table Association results for single markers at allelic model. (DOCX)

S5 Table Association results for single markers at allelic model in female samples. (DOCX)

S6 Table Association results for single markers at allelic model in male samples. (DOCX)

S7 Table Candidate SNP´s frequencies in European population. (DOCX)

S8 Table Mean values and standard deviation (SD) of the psychometric characteristics across ages for dyslexia and dyslexia-control samples. (DOCX)

S9 Table Mean values and standard deviation (SD) of the psychometric characteristics across ages for ADHD and ADHD-control samples. (DOCX)

S10 Table Mean values and standard deviation (SD) of the psychometric characteristics across ages for comorbid and comorbid-control samples. (DOCX)

S1 File Description of cognitive tasks. (DOCX)

S1 Fig Principal component analysis. Graphic representation of the two principal components obtained through the PLINK program for the cases and controls of dyslexia, ADHD and comorbidity after whole genome genotyping with OmniExpress Beadchips (Illumina Inc.). Green dots = Dyslexia; grey = Dyslexia controls; pink = ADHD; black = ADHD controls; blue = comorbids; yellow = Comorbid controls.
(PDF)

S1 Dataset Contains the SNP genotyping raw data for each performed comparative as *.ped and *.map files, and a subfolder named “Freq_two locus” containing the frequency of the composite genotypes (two by two). (RAR)
