# Correction: Genetic association study of dyslexia and ADHD candidate genes in a Spanish cohort: Implications of comorbid samples

**DOI:** 10.1371/journal.pone.0209718

**Published:** 2018-12-19

**Authors:** Mirian Sánchez-Morán, Juan Andrés Hernández, Jon Andoni Duñabeitia, Adelina Estévez, Laura Bárcena, Aintzane González-Lahera, María Teresa Bajo, Luis J. Fuentes, Ana M. Aransay, Manuel Carreiras

There are errors in the Author Contributions. The correct contributions are: Conceptualization: AMA MC. Formal analysis: MSM AMA JAH MC LB AGL. Funding acquisition: MC AMA. Investigation: MSM JAD AE MTB LJF AMA MC. Methodology: MSM AMA JAH MC. Project administration: MC AMA. Supervision: AMA MC. Writing – original draft: MSM AMA. Writing – review and editing: MSM AMA MC.
