# Supplementary material for: Genetic association study of dyslexia and ADHD candidate genes in a Spanish cohort: Implications of comorbid samples
Source: PLoS One. 2018 Oct 31;13(10):e0206431. doi: 10.1371/journal.pone.0206431 (PMC6209299; doi:10.1371/journal.pone.0206431)
Supplement: S1 Table — (DOCX) [file pone.0206431.s001.docx]

**S1 Table**. Association results for single markers at different genetic models, considering the whole population. The numbers of individuals for each studied population are detailed below each comparative.

|  |  |  |  |  |  | **Dys** | **ADHD** | **Com** | **Com** | **Com** | **Dys+Com** | **ADHD+Com** |
| --- | --- | --- | --- | --- | --- | --- | --- | --- | --- | --- | --- | --- |
| **GENE** | **CHR** | **SNP** | **A1** | **A2** | **TEST** | **Ctr__Dys_** | **Ctr__ADHD_** | **Ctr__Dys_** | **Ctr__ADHD_** | **Ctr__Com_** | **Ctr__Dys_** | **Ctr__ADHD_** |
| ***DCDC2*** | 6 | rs2274305 | T | C | GENOTYPIC | 0.6234 | 0.8522 | NA | NA | NA | 0.5365 | 0.9882 |
|  |  |  |  |  | ALLELIC | 0.9424 | 0.5819 | 0.1969 | 0.3516 | 0.3228 | 0.5935 | 0.8998 |
|  |  |  |  |  | DOMINANT | 0.6793 | 0.6026 | NA | NA | NA | 0.9925 | 0.8780 |
|  |  |  |  |  | RECESSIVE | 0.4821 | 0.7123 | NA | NA | NA | 0.2913 | 0.9727 |
| ***KIAA0319*** | 6 | rs4504469 | T | C | GENOTYPIC | 0.2290 | 0.7987 | NA | NA | NA | **0.0267** | 0.2339 |
|  |  |  |  |  | ALLELIC | **0.0913** | 0.5377 | **0.0025** | **0.0032** | **0.0101** | **0.0084** | **0.0953** |
|  |  |  |  |  | DOMINANT | 0.1597 | 0.5055 | NA | NA | NA | **0.0299** | 0.1249 |
|  |  |  |  |  | RECESSIVE | 0.1692 | 0.7824 | NA | NA | NA | **0.0289** | 0.2442 |
| ***FOXP2*** | 7 | rs12533005 | C | G | GENOTYPIC | 0.3324 | 0.2577 | **0.0323** | **0.0045** | **0.0126** | **0.0716** | **0.0795** |
|  |  |  |  |  | ALLELIC | 0.1277 | 0.6375 | **0.0077** | **0.0015** | **0.0096** | **0.0191** | 0.1058 |
|  |  |  |  |  | DOMINANT | 0.1843 | 0.6599 | **0.0160** | **0.0158** | **0.0988** | **0.0370** | 0.5989 |
|  |  |  |  |  | RECESSIVE | 0.2710 | 0.1744 | **0.0665** | **0.0040** | **0.0049** | **0.0985** | **0.0251** |
| ***DYX1C1*** | 15 | rs57809907 | A | C | GENOTYPIC | 0.7643^a^ | NA | NA | NA | NA | 0.9043^a^ | 0.7464 |
|  |  |  |  |  | ALLELIC | 0.9353^a^ | 0.9315 | 0.3694^a^ | 0.1902 | 0.1516 | 0.6887^a^ | 0.6654 |
|  |  |  |  |  | DOMINANT | 0.7542^a^ | NA | NA | NA | NA | 0.6668^a^ | 0.8501 |
|  |  |  |  |  | RECESSIVE | 0.6209^a^ | NA | NA | NA | NA | 0.96^a^ | 0.4457 |
| ***DBH*** | 9 | rs1611115 | T | C | GENOTYPIC | 0.7604 | 0.3410 | NA | NA | NA | 0.6355 | 0.3361 |
|  |  |  |  |  | ALLELIC | 0.5746 | 0.1664 | 0.8218 | 0.9194 | 0.1763 | 0.5555 | 0.2140 |
|  |  |  |  |  | DOMINANT | 0.7165 | 0.2511 | NA | NA | NA | 0.7640 | 0.3584 |
|  |  |  |  |  | RECESSIVE | 0.4717 | 0.2339 | NA | NA | NA | 0.3415 | 0.1742 |
| ***COMT1*** | 22 | rs4680 | A | G | GENOTYPIC | **0.0621** | 0.6131 | **0.01813** | 0.1732 | 0.3996 | **0.0193** | 0.5011 |
|  |  |  |  |  | ALLELIC | 0.4648 | 0.5494 | **0.0747** | **0.0888** | 0.1361 | 0.9883 | 0.2416 |
|  |  |  |  |  | DOMINANT | 0.5526 | 0.9452 | **0.0065** | **0.0612** | 0.2127 | 0.1287 | 0.4388 |
|  |  |  |  |  | RECESSIVE | **0.0464** | 0.3460 | 0.9522 | 0.4583 | 0.3055 | **0.0750** | 0.2671 |
| ***MAOA*** | 23 | rs6323 | G | T | GENOTYPIC | 0.6025 | 0.1882 | NA | NA | NA | 0.2812 | **0.0744** |
|  |  |  |  |  | ALLELIC | 0.6392 | 0.1597 | 0.3306 | 0.5030 | 0.5486 | 0.4623 | 0.1362 |
|  |  |  |  |  | DOMINANT | 0.9226 | 0.3840 | NA | NA | NA | 0.8922 | 0.4903 |
|  |  |  |  |  | RECESSIVE | 0.3246 | **0.0738** | NA | NA | NA | 0.1205 | **0.0227** |
| **Nº Cas** | | | | | | 241 | 187 | 45 | 45 | 45 | 286 | 232 |
| **Nº Ctr** | | | | | | 1197 | 514 | 1199 | 514 | 106 | 1197 | 514 |

Abbreviations: Chr=chromosome, A1=allele 1, A2=allele 2. The grey square shows the case groups in the superior line and the control groups in the inferior one. Dys=dyslexia samples, ADHD=Attention Deficit Hyperactivity Disorder samples, Com=Comorbid samples, Ctr__Dys_=dyslexia controls, Ctr__ADHD_=ADHD controls, Ctr__com_= Comorbid controls, a=not in Hardy-Weinberg equilibrium. Nº Cas= number of case samples, Nº Ctr=number of control samples, Nº Males Cas= number of male-case samples, Nº Males Ctr= number of male-control samples Nº Females Cas=number of female-case samples, Nº Females Ctr=number of female-control samples. Significance values <0.05 are represented in red. Significance trend values<0.1 are represented in bold.
