# Supplementary material for: Genetic association study of dyslexia and ADHD candidate genes in a Spanish cohort: Implications of comorbid samples
Source: PLoS One. 2018 Oct 31;13(10):e0206431. doi: 10.1371/journal.pone.0206431 (PMC6209299; doi:10.1371/journal.pone.0206431)
Supplement: S2 Table — (DOCX) [file pone.0206431.s002.docx]

**S2 Table**. Association results for single markers at different genetic models, considering only female samples. The numbers of individuals for each studied population are detailed below each comparative.

|  |  |  |  |  |  | **Dys** | **ADHD** | **Com** | **Com** | **Com** | **Dys+Com** | **ADHD+Com** |
| --- | --- | --- | --- | --- | --- | --- | --- | --- | --- | --- | --- | --- |
| **GENE** | **CHR** | **SNP** | **A1** | **A2** | **TEST** | **Ctr__Dys_** | **Ctr__ADHD_** | **Ctr__Dys_** | **Ctr__ADHD_** | **Ctr__com_** | **Ctr__Dys_** | **Ctr__ADHD_** |
| ***DCDC2*** | 6 | rs2274305 | T | C | GENOTYPIC | 0.8234 | 0.8886 | NA | NA | NA | 0.9480 | 0.7872 |
|  |  |  |  |  | ALLELIC | 0.5349 | 0.8346 | 0.3921 | 0.5510 | 0.8546 | 0.7526 | 0.6781 |
|  |  |  |  |  | DOMINANT | 0.6121 | 0.9897 | NA | NA | NA | 0.7511 | 0.8980 |
|  |  |  |  |  | RECESSIVE | 0.6110 | 0.6449 | NA | NA | NA | 0.8584 | 0.4916 |
| ***KIAA0319*** | 6 | rs4504469 | T | C | GENOTYPIC | 0.2775 | 0.6827 | NA | NA | NA | 0.1060 | 0.6449 |
|  |  |  |  |  | ALLELIC | 0.1183 | 0.8892 | **0.0493** | **0.0767** | **0.0991** | **0.0387** | 0.5713 |
|  |  |  |  |  | DOMINANT | 0.1821 | 0.7938 | NA | NA | NA | **0.0754** | 0.3987 |
|  |  |  |  |  | RECESSIVE | 0.2127 | 0.4737 | NA | NA | NA | 0.1024 | 0.8923 |
| ***FOXP2*** | 7 | rs12533005 | C | G | GENOTYPIC | 0.4791 | 0.4436 | NA | NA | NA | 0.3779 | 0.2442 |
|  |  |  |  |  | ALLELIC | 0.2168 | 0.5500 | 0.3825 | 0.1914 | 0.2462 | 0.1557 | 0.3030 |
|  |  |  |  |  | DOMINANT | 0.3886 | 0.9510 | NA | NA | NA | 0.3241 | 0.8412 |
|  |  |  |  |  | RECESSIVE | 0.2657 | 0.2348 | NA | NA | NA | 0.1993 | 0.1004 |
| ***DYX1C1*** | 15 | rs57809907 | A | C | GENOTYPIC | NA | NA | NA | NA | 0.5711 | NA | NA |
|  |  |  |  |  | ALLELIC | 0.5101^a^ | 0.3863 | 0.7584^a^ | 0.9201 | NA | 0.4776^a^ | 0.4238 |
|  |  |  |  |  | DOMINANT | NA | NA | NA | NA | NA | NA | NA |
|  |  |  |  |  | RECESSIVE | NA | NA | NA | NA | NA | NA | NA |
| ***DBH*** | 9 | rs1611115 | T | C | GENOTYPIC | NA | NA | NA | NA | NA | 0.6081 | NA |
|  |  |  |  |  | ALLELIC | 0.2629 | 0.8947 | 0.8169 | 0.6856 | 0.2350 | 0.3318 | 0.7851 |
|  |  |  |  |  | DOMINANT | NA | NA | NA | NA | NA | 0.3859 | NA |
|  |  |  |  |  | RECESSIVE | NA | NA | NA | NA | NA | 0.4801 | NA |
| ***COMT*** | 22 | rs4680 | A | G | GENOTYPIC | 0.3253 | 0.3385 | NA | NA | NA | 0.1508 | 0.3028 |
|  |  |  |  |  | ALLELIC | 0.9215 | 0.1491 | 0.2215 | 0.4225 | 0.7812 | 0.7755 | 0.1135 |
|  |  |  |  |  | DOMINANT | 0.4612 | 0.3032 | NA | NA | NA | 0.1931 | 0.1741 |
|  |  |  |  |  | RECESSIVE | 0.3024 | 0.1796 | NA | NA | NA | 0.3202 | 0.2439 |
| ***MAOA*** | 23 | rs6323 | G | T | GENOTYPIC | 0.6025 | 0.1882 | NA | NA | NA | 0.2812 | **0.0744** |
|  |  |  |  |  | ALLELIC | 0.6392 | 0.1597 | 0.3306 | 0.5030 | 0.5486 | 0.4623 | 0.1362 |
|  |  |  |  |  | DOMINANT | 0.9226 | 0.3840 | NA | NA | NA | 0.8922 | 0.4903 |
|  |  |  |  |  | RECESSIVE | 0.3246 | 0.0738 | NA | NA | NA | 0.1205 | **0.0227** |
| **Nº Cas** | | | | | | 125 | 69 | 16 | 16 | 16 | 141 | 85 |
| **Nº Ctr** | | | | | | 633 | 255 | 634 | 255 | 39 | 633 | 255 |

Abbreviations: Chr=chromosome, A1=allele 1, A2=allele 2. The grey square shows the case groups in the superior line and the control groups in the inferior one. Dys=dyslexia samples, ADHD=Attention Deficit Hyperactivity Disorder samples, Com=Comorbid samples, Ctr__Dys_=dyslexia controls, Ctr__ADHD_=ADHD controls, Ctr__com_=Comorbid controls, a=not in Hardy-Weinberg equilibrium. Nº Cas=number of case samples, Nº Ctr=number of control samples. Significance values <0.05 are represented in red. Significance trend values<0.1 are represented in bold.
