# Supplementary material for: Genetic association study of dyslexia and ADHD candidate genes in a Spanish cohort: Implications of comorbid samples
Source: PLoS One. 2018 Oct 31;13(10):e0206431. doi: 10.1371/journal.pone.0206431 (PMC6209299; doi:10.1371/journal.pone.0206431)
Supplement: S3 Table — (DOCX) [file pone.0206431.s003.docx]

**S3 Table**. Association results for single markers at different genetic models, considering only male samples. The numbers of individuals for each studied population are detailed below each comparative.

|  |  |  |  |  |  | **Dys** | **ADHD** | **Com** | **Com** | **Com** | **Dys+Com** | **ADHD+Com** |
| --- | --- | --- | --- | --- | --- | --- | --- | --- | --- | --- | --- | --- |
| **GENE** | **CHR** | **SNP** | **A1** | **A2** | **TEST** | **Ctr__Dys_** | **Ctr__ADHD_** | **Ctr__Dys_** | **Ctr__ADHD_** | **Ctr__com_** | **Ctr__Dys_** | **Ctr__ADHD_** |
| ***DCDC2*** | 6 | rs2274305 | T | C | GENOTYPIC | 0.2454 | 0.7432 | NA | NA | NA | 0.2391 | 0.9168 |
|  |  |  |  |  | ALLELIC | 0.4532 | 0.4579 | 0.3211 | 0.4482 | 0.1718 | 0.2872 | 0.7189 |
|  |  |  |  |  | DOMINANT | 0.9483 | 0.5639 | NA | NA | NA | 0.7542 | 0.8253 |
|  |  |  |  |  | RECESSIVE | 0.1154 | 0.5059 | NA | NA | NA | **0.0933** | 0.6856 |
| ***KIAA0319*** | 6 | rs4504469 | T | C | GENOTYPIC | 0.7107 | 0.5568 | NA | NA | NA | 0.2327 | 0.1631 |
|  |  |  |  |  | ALLELIC | 0.4170 | 0.3164 | **0.0216** | **0.0147** | **0.0473** | 0.0973 | **0.0740** |
|  |  |  |  |  | DOMINANT | 0.5240 | 0.4552 | NA | NA | NA | 0.2004 | 0.1629 |
|  |  |  |  |  | RECESSIVE | 0.4812 | 0.3316 | NA | NA | NA | 0.1387 | **0.0988** |
| ***FOXP2*** | 7 | rs12533005 | C | G | GENOTYPIC | 0.6004 | 0.5186 | NA | NA | NA | 0.1497 | 0.3981 |
|  |  |  |  |  | ALLELIC | 0.3699 | 0.9234 | **0.0114** | **0.0051** | **0.0184** | **0.0688** | 0.3342 |
|  |  |  |  |  | DOMINANT | 0.3139 | 0.4800 | NA | NA | NA | **0.0557** | 0.7828 |
|  |  |  |  |  | RECESSIVE | 0.6673 | 0.5350 | NA | NA | NA | 0.3084 | 0.1783 |
| ***DYX1C1*** | 15 | rs57809907 | A | C | GENOTYPIC | NA | NA | NA | NA | NA | 0.1418 | 0.4110 |
|  |  |  |  |  | ALLELIC | 0.3980 | 0.5029 | 0.1386 | **0.0707** | 0.1810 | 0.1819 | 0.2166 |
|  |  |  |  |  | DOMINANT | NA | NA | NA | NA | NA | 0.5006 | 0.4154 |
|  |  |  |  |  | RECESSIVE | NA | NA | NA | NA | NA | **0.0481** | 0.1976 |
| ***DBH*** | 9 | rs1611115 | T | C | GENOTYPIC | 0.8241 | 0.1202 | NA | NA | NA | 0.6389 | 0.2240 |
|  |  |  |  |  | ALLELIC | 0.6977 | 0.0758 | 0.7313 | 0.6911 | 0.4261 | 0.8409 | **0.0917** |
|  |  |  |  |  | DOMINANT | 0.5951 | 0.0443 | NA | NA | NA | 0.6133 | **0.0844** |
|  |  |  |  |  | RECESSIVE | 0.8732 | 0.8519 | NA | NA | NA | 0.5293 | 0.5414 |
| ***COMT1*** | 22 | rs4680 | A | G | GENOTYPIC | 0.1611 | 0.6118 | 0.1726 | 0.2564 | 0.3152 | 0.1274 | 0.6310 |
|  |  |  |  |  | ALLELIC | 0.3410 | 0.9618 | 0.1960 | **0.0860** | **0.0954** | 0.7593 | 0.4943 |
|  |  |  |  |  | DOMINANT | 0.9294 | 0.6173 | **0.0671** | 0.1166 | 0.2623 | 0.4086 | 0.8683 |
|  |  |  |  |  | RECESSIVE | **0.0748** | 0.5486 | 0.8525 | 0.2720 | 0.1534 | 0.1364 | 0.3493 |
| ***MAOA*** | 23 | rs6323 | G | T | GENOTYPIC | NA | NA | NA | NA | NA | NA | NA |
|  |  |  |  |  | ALLELIC | NA | NA | NA | NA | NA | NA | NA |
|  |  |  |  |  | DOMINANT | NA | NA | NA | NA | NA | NA | NA |
|  |  |  |  |  | RECESSIVE | NA | NA | NA | NA | NA | NA | NA |
| **Nº Cas** | | | | | | 116 | 118 | 29 | 29 | 29 | 145 | 147 |
| **Nº Ctr** | | | | | | 564 | 259 | 565 | 259 | 67 | 564 | 259 |

Abbreviations: Chr=chromosome, A1=allele 1, A2=allele 2. The grey square shows the case groups in the superior line and the control groups in the inferior one. Dys=dyslexia samples, ADHD=Attention Deficit Hyperactivity Disorder samples, Com=Comorbid samples, Ctr__Dys_=dyslexia controls, Ctr__ADHD_=ADHD controls, Ctr__com_=Comorbid controls. Nº Cas= number of case samples, Nº Ctr=number of control samples. Significance values <0.05 are represented in red. Significance trend values<0.1 are represented in bold.
