# Supplementary material for: Genetic association study of dyslexia and ADHD candidate genes in a Spanish cohort: Implications of comorbid samples
Source: PLoS One. 2018 Oct 31;13(10):e0206431. doi: 10.1371/journal.pone.0206431 (PMC6209299; doi:10.1371/journal.pone.0206431)
Supplement: S4 Table — (DOCX) [file pone.0206431.s004.docx]

**S4 Table**. Basic case/control association analysis results for single markers considering the whole population (allelic model).

| **GEN** | CHR | SNP | BP | A1 | F_A | F_U | A2 | CHISQ | P | OR | SE | L95 | U95 | **TEST DEFINITION** |
| --- | --- | --- | --- | --- | --- | --- | --- | --- | --- | --- | --- | --- | --- | --- |
| DCDC2 | 6 | rs2274305 | 24291203 | T | 0.3776 | 0.3897 | C | 0.2849 | 0.5935 | 0.9501 | 0.09589 | 0.7873 | 1.147 | **Dys+Com vs Ctr_Dys** |
| KIAA0319 | 6 | rs4504469 | 24588884 | T | 0.2965 | 0.3548 | C | 6.941 | 0.008425 | 0.7664 | 0.1012 | 0.6285 | 0.9345 |  |
| FOXP2 | 7 | rs12533005 | 114056055 | C | 0.5227 | 0.4683 | G | 5.49 | 0.01913 | 1.244 | 0.09319 | 1.036 | 1.493 |  |
| DBH | 9 | rs1611115 | 136500515 | T | 0.2098 | 0.2211 | C | 0.3476 | 0.5555 | 0.935 | 0.114 | 0.7478 | 1.169 |  |
| DYX1C1 | 15 | rs57809907 | 55722882 | A | 0.1095 | 0.1038 | C | 0.1605 | 0.6887 | 1.062 | 0.1505 | 0.7908 | 1.427 |  |
| COMT1 | 22 | rs4680 | 19951271 | A | 0.451 | 0.4507 | G | 0.0002141 | 0.9883 | 1.001 | 0.09353 | 0.8336 | 1.203 |  |
| MAOA | 23 | rs6323 | 43591036 | G | 0.2623 | 0.2403 | T | 0.9078 | 0.3407 | 1.124 | 0.1229 | 0.8835 | 1.43 |  |
| DCDC2 | 6 | rs2274305 | 24291203 | T | 0,388 | 0,3897 | C | 0,005212 | 0,9424 | 0,9926 | 0,1024 | 0,8121 | 1,213 | **Dys vs Ctr_Dys** |
| KIAA0319 | 6 | rs4504469 | 24588884 | T | 0,3146 | 0,3548 | C | 2,851 | **0,0913** | 0,8346 | 0,1072 | 0,6764 | 1,03 |  |
| FOXP2 | 7 | rs12533005 | 114056055 | C | 0,5062 | 0,4683 | G | 2,32 | 0,1277 | 1,164 | 0,09989 | 0,9572 | 1,416 |  |
| DBH | 9 | rs1611115 | 136500515 | T | 0,2095 | 0,2211 | C | 0,3151 | 0,5746 | 0,9336 | 0,1224 | 0,7346 | 1,187 |  |
| DYX1C1 | 15 | rs57809907 | 55722882 | A | 0,105 | 0,1038 | C | 0,006588 | 0,9353 | 1,013 | 0,164 | 0,7349 | 1,397 |  |
| COMT1 | 22 | rs4680 | 19951271 | A | 0,4689 | 0,4507 | G | 0,5344 | 0,4648 | 1,076 | 0,1001 | 0,8842 | 1,309 |  |
| MAOA | 23 | rs6323 | 43591036 | G | 0,2678 | 0,2403 | T | 1,245 | 0,2645 | 1,156 | 0,1301 | 0,8959 | 1,492 |  |
| DCDC2 | 6 | rs2274305 | 24291203 | T | 0,3222 | 0,3891 | C | 1,635 | 0,2011 | 0,7465 | 0,2294 | 0,4762 | 1,17 | **Com vs Ctr_Dys** |
| KIAA0319 | 6 | rs4504469 | 24588884 | T | 0,2 | 0,3546 | C | 9,129 | 0,002516 | 0,4549 | 0,267 | 0,2696 | 0,7677 |  |
| FOXP2 | 7 | rs12533005 | 114056055 | C | 0,6111 | 0,4687 | G | 7,054 | 0,007909 | 1,781 | 0,2201 | 1,157 | 2,742 |  |
| DBH | 9 | rs1611115 | 136500515 | T | 0,2111 | 0,2208 | C | 0,04712 | 0,8282 | 0,9445 | 0,263 | 0,5641 | 1,581 |  |
| DYX1C1 | 15 | rs57809907 | 55722882 | A | 0,1333 | 0,1036 | C | 0,8165 | 0,3662 | 1,331 | 0,3173 | 0,7145 | 2,479 |  |
| COMT1 | 22 | rs4680 | 19951271 | A | 0,3556 | 0,4512 | G | 3,21 | **0,0732** | 0,671 | 0,224 | 0,4326 | 1,041 |  |
| MAOA | 23 | rs6323 | 43591036 | G | 0,2295 | 0,24 | T | 0,03581 | 0,8499 | 0,9431 | 0,3094 | 0,5143 | 1,729 |  |
| DCDC2 | 6 | rs2274305 | 24291203 | T | 0.3222 | 0.3716 | C | 0.8678 | 0.3516 | 0.804 | 0.2346 | 0.5076 | 1.273 | **Com vs Ctr_ADHD** |
| KIAA0319 | 6 | rs4504469 | 24588884 | T | 0.2 | 0.3538 | C | 8.705 | 0.003174 | 0.4566 | 0.2715 | 0.2682 | 0.7774 |  |
| FOXP2 | 7 | rs12533005 | 114056055 | C | 0.6111 | 0.4377 | G | 10.04 | 0.001529 | 2.018 | 0.2252 | 1.298 | 3.138 |  |
| DBH | 9 | rs1611115 | 136500515 | T | 0.2111 | 0.2157 | C | 0.01025 | 0.9194 | 0.9731 | 0.2693 | 0.5741 | 1.65 |  |
| DYX1C1 | 15 | rs57809907 | 55722882 | A | 0.1333 | 0.09118 | C | 1.716 | 0.1902 | 1.533 | 0.3286 | 0.8053 | 2.92 |  |
| COMT1 | 22 | rs4680 | 19951271 | A | 0.3556 | 0.4484 | G | 2.896 | **0.08877** | 0.6786 | 0.229 | 0.4332 | 1.063 |  |
| MAOA | 23 | rs6323 | 43591036 | G | 0.2295 | 0.2565 | T | 0.2172 | 0.6412 | 0.8634 | 0.3155 | 0.4652 | 1.602 |  |
| DCDC2 | 6 | rs2274305 | 24291203 | T | 0,3222 | 0,3821 | C | 0,9774 | 0,3228 | 0,7689 | 0,2662 | 0,4563 | 1,295 | **Com vs Ctr_Com** |
| KIAA0319 | 6 | rs4504469 | 24588884 | T | 0,2 | 0,3491 | C | 6,626 | 0,01005 | 0,4662 | 0,3003 | 0,2588 | 0,8399 |  |
| FOXP2 | 7 | rs12533005 | 114056055 | C | 0,6111 | 0,4481 | G | 6,715 | 0,009563 | 1,935 | 0,2566 | 1,17 | 3,2 |  |
| DBH | 9 | rs1611115 | 136500515 | T | 0,2111 | 0,1476 | C | 1,829 | 0,1763 | 1,545 | 0,3234 | 0,8199 | 2,912 |  |
| DYX1C1 | 15 | rs57809907 | 55722882 | A | 0,1333 | 0,08019 | C | 2,056 | 0,1516 | 1,765 | 0,4001 | 0,8055 | 3,866 |  |
| COMT1 | 22 | rs4680 | 19951271 | A | 0,3556 | 0,4481 | G | 2,221 | 0,1361 | 0,6795 | 0,2599 | 0,4083 | 1,131 |  |
| MAOA | 23 | rs6323 | 43591036 | G | 0,2295 | 0,2759 | T | 0,477 | 0,4898 | 0,7819 | 0,3567 | 0,3886 | 1,573 |  |
| DCDC2 | 6 | rs2274305 | 24291203 | T | 0.375 | 0.3716 | C | 0.01585 | 0.8998 | 1.015 | 0.1156 | 0.809 | 1.273 | **ADHD+Com vs Ctr_ADHD** |
| KIAA0319 | 6 | rs4504469 | 24588884 | T | 0.3095 | 0.3538 | C | 2.783 | **0.09528** | 0.8188 | 0.12 | 0.6472 | 1.036 |  |
| FOXP2 | 7 | rs12533005 | 114056055 | C | 0.4828 | 0.4377 | G | 2.616 | 0.1058 | 1.199 | 0.1122 | 0.9622 | 1.494 |  |
| DBH | 9 | rs1611115 | 136500515 | T | 0.1875 | 0.2157 | C | 1.544 | 0.214 | 0.8392 | 0.1412 | 0.6363 | 1.107 |  |
| DYX1C1 | 15 | rs57809907 | 55722882 | A | 0.09825 | 0.09118 | C | 0.187 | 0.6654 | 1.086 | 0.191 | 0.747 | 1.579 |  |
| COMT1 | 22 | rs4680 | 19951271 | A | 0.4159 | 0.4484 | G | 1.371 | 0.2416 | 0.8759 | 0.1132 | 0.7017 | 1.093 |  |
| MAOA | 23 | rs6323 | 43591036 | G | 0.2839 | 0.2565 | T | 0.866 | 0.3521 | 1.149 | 0.1495 | 0.8573 | 1.54 |  |
| DCDC2 | 6 | rs2274305 | 24291203 | T | 0,3877 | 0,3716 | C | 0,3032 | 0,5819 | 1,071 | 0,1242 | 0,8394 | 1,366 | **ADHD vs Ctr_ADHD** |
| KIAA0319 | 6 | rs4504469 | 24588884 | T | 0,336 | 0,3538 | C | 0,3798 | 0,5377 | 0,9243 | 0,1277 | 0,7196 | 1,187 |  |
| FOXP2 | 7 | rs12533005 | 114056055 | C | 0,4519 | 0,4377 | G | 0,222 | 0,6375 | 1,059 | 0,1214 | 0,8346 | 1,343 |  |
| DBH | 9 | rs1611115 | 136500515 | T | 0,1818 | 0,2157 | C | 1,915 | 0,1664 | 0,8081 | 0,1542 | 0,5973 | 1,093 |  |
| DYX1C1 | 15 | rs57809907 | 55722882 | A | 0,08967 | 0,09118 | C | 0,007397 | 0,9315 | 0,9819 | 0,2124 | 0,6475 | 1,489 |  |
| COMT1 | 22 | rs4680 | 19951271 | A | 0,4305 | 0,4484 | G | 0,3585 | 0,5494 | 0,9297 | 0,1218 | 0,7322 | 1,18 |  |
| MAOA | 23 | rs6323 | 43591036 | G | 0,2969 | 0,2565 | T | 1,6 | 0,2059 | 1,224 | 0,1598 | 0,8947 | 1,674 |  |

Abbreviations: Chr=chromosome, BP=base pair, A1=allele 1, F_A=Frequency in affected individuals, F_U=Frequency in unaffected individuals, A2=allele 2.OR=Estimated odds ratio for A1, L95=Lower bound of 95% confidence interval for odds ratio, U95= Uper bound of 95% confidence interval for odds ratio, Dys=dyslexia samples, ADHD=Attention Deficit Hyperactivity Disorder samples, Com=Comorbid samples, Ctr__Dys_=dyslexia controls, Ctr__ADHD_=ADHD controls, Ctr__com_=Comorbid controls. Significance values <0.05 are represented in red. Significance trend values<0.1 are represented in bold.
