# Supplementary material for: Genetic association study of dyslexia and ADHD candidate genes in a Spanish cohort: Implications of comorbid samples
Source: PLoS One. 2018 Oct 31;13(10):e0206431. doi: 10.1371/journal.pone.0206431 (PMC6209299; doi:10.1371/journal.pone.0206431)
Supplement: S5 Table — (DOCX) [file pone.0206431.s005.docx]

**S5 Table**. Basic case/control association analysis results for single markers considering only female samples (allelic model).

| **GEN** | **CHR** | **SNP** | **BP** | **A1** | **F_A** | **F_U** | **A2** | **CHISQ** | **P** | **OR** | **SE** | **L95** | **U95** | **TEST DEFINITION** |
| --- | --- | --- | --- | --- | --- | --- | --- | --- | --- | --- | --- | --- | --- | --- |
| DCDC2 | 6 | rs2274305 | 24291203 | T | 0.3972 | 0.387 | C | 0.09933 | 0.7526 | 1.043 | 0.1347 | 0.8013 | 1.359 | **Dys+Com vs Ctr_Dys** |
| KIAA0319 | 6 | rs4504469 | 24588884 | T | 0.2908 | 0.3555 | C | 4.276 | 0.03865 | 0.7435 | 0.1437 | 0.561 | 0.9853 |  |
| FOXP2 | 7 | rs12533005 | 114056055 | C | 0.5 | 0.4534 | G | 2.015 | 0.1557 | 1.206 | 0.1318 | 0.9311 | 1.561 |  |
| DBH | 9 | rs1611115 | 136500515 | T | 0.2057 | 0.2325 | C | 0.9417 | 0.3318 | 0.8548 | 0.1618 | 0.6226 | 1.174 |  |
| DYX1C1 | 15 | rs57809907 | 55722882 | A | 0.09643 | 0.111 | C | 0.5044 | 0.4776 | 0.8545 | 0.2215 | 0.5535 | 1.319 |  |
| COMT1 | 22 | rs4680 | 19951271 | A | 0.4433 | 0.4526 | G | 0.08133 | 0.7755 | 0.9629 | 0.1325 | 0.7427 | 1.248 |  |
| MAOA | 23 | rs6323 | 43591036 | G | 0.2589 | 0.2381 | T | 0.5404 | 0.4623 | 1.117 | 0.1511 | 0.831 | 1.503 |  |
| DCDC2 | 6 | rs2274305 | 24291203 | T | 0,408 | 0,387 | C | 0,3851 | 0,5349 | 1,091 | 0,141 | 0,8279 | 1,439 | **Dys vs Ctr_Dys** |
| KIAA0319 | 6 | rs4504469 | 24588884 | T | 0,304 | 0,3555 | C | 2,439 | 0,1183 | 0,792 | 0,1495 | 0,5909 | 1,062 |  |
| FOXP2 | 7 | rs12533005 | 114056055 | C | 0,496 | 0,4534 | G | 1,525 | 0,2168 | 1,186 | 0,1385 | 0,9043 | 1,557 |  |
| DBH | 9 | rs1611115 | 136500515 | T | 0,2 | 0,2325 | C | 1,253 | 0,2629 | 0,8253 | 0,1716 | 0,5896 | 1,155 |  |
| DYX1C1 | 15 | rs57809907 | 55722882 | A | 0,09677 | 0,111 | C | 0,4339 | 0,5101 | 0,8579 | 0,2329 | 0,5435 | 1,354 |  |
| COMT1 | 22 | rs4680 | 19951271 | A | 0,456 | 0,4526 | G | 0,009701 | 0,9215 | 1,014 | 0,139 | 0,7721 | 1,331 |  |
| MAOA | 23 | rs6323 | 43591036 | G | 0,252 | 0,2381 | T | 0,2198 | 0,6392 | 1,078 | 0,1599 | 0,7878 | 1,475 |  |
| DCDC2 | 6 | rs2274305 | 24291203 | T | 0,3125 | 0,3864 | C | 0,7209 | 0,3959 | 0,7217 | 0,3857 | 0,3389 | 1,537 | **Com vs Ctr_Dys** |
| KIAA0319 | 6 | rs4504469 | 24588884 | T | 0,1875 | 0,3557 | C | 3,873 | 0,04908 | 0,418 | 0,4567 | 0,1708 | 1,023 |  |
| FOXP2 | 7 | rs12533005 | 114056055 | C | 0,5312 | 0,4535 | G | 0,7614 | 0,3829 | 1,366 | 0,3587 | 0,6762 | 2,759 |  |
| DBH | 9 | rs1611115 | 136500515 | T | 0,25 | 0,2321 | C | 0,05593 | 0,813 | 1,103 | 0,4137 | 0,4902 | 2,481 |  |
| DYX1C1 | 15 | rs57809907 | 55722882 | A | 0,09375 | 0,1108 | C | 0,09284 | 0,7606 | 0,8298 | 0,6131 | 0,2495 | 2,76 |  |
| COMT1 | 22 | rs4680 | 19951271 | A | 0,3438 | 0,4535 | G | 1,518 | 0,218 | 0,6313 | 0,3764 | 0,3019 | 1,32 |  |
| MAOA | 23 | rs6323 | 43591036 | G | 0,3125 | 0,2378 | T | 0,957 | 0,3279 | 1,457 | 0,3871 | 0,6825 | 3,112 |  |
| DCDC2 | 6 | rs2274305 | 24291203 | T | 0.3125 | 0.3647 | C | 0.3555 | 0.551 | 0.7918 | 0.3923 | 0.367 | 1.708 | **Com vs Ctr_ADHD** |
| KIAA0319 | 6 | rs4504469 | 24588884 | T | 0.1875 | 0.3392 | C | 3.133 | **0.0767** | 0.4495 | 0.4625 | 0.1816 | 1.113 |  |
| FOXP2 | 7 | rs12533005 | 114056055 | C | 0.5312 | 0.4137 | G | 1.707 | 0.1914 | 1.606 | 0.3655 | 0.7846 | 3.287 |  |
| DBH | 9 | rs1611115 | 136500515 | T | 0.25 | 0.2194 | C | 0.1639 | 0.6856 | 1.186 | 0.4221 | 0.5186 | 2.713 |  |
| DYX1C1 | 15 | rs57809907 | 55722882 | A | 0.09375 | 0.09921 | C | 0.01005 | 0.9201 | 0.9393 | 0.6245 | 0.2762 | 3.194 |  |
| COMT1 | 22 | rs4680 | 19951271 | A | 0.3438 | 0.4157 | G | 0.6435 | 0.4225 | 0.7363 | 0.3829 | 0.3476 | 1.559 |  |
| MAOA | 23 | rs6323 | 43591036 | G | 0.3125 | 0.2588 | T | 0.4487 | 0.503 | 1.302 | 0.3946 | 0.6007 | 2.821 |  |
| DCDC2 | 6 | rs2274305 | 24291203 | T | 0,3125 | 0,2949 | C | 0,03358 | 0,8546 | 1,087 | 0,4551 | 0,4455 | 2,652 | **Com vs Ctr_Com** |
| KIAA0319 | 6 | rs4504469 | 24588884 | T | 0,1875 | 0,3462 | C | 2,72 | **0,09911** | 0,4359 | 0,5116 | 0,1599 | 1,188 |  |
| FOXP2 | 7 | rs12533005 | 114056055 | C | 0,5312 | 0,4103 | G | 1,345 | 0,2462 | 1,629 | 0,4225 | 0,7118 | 3,729 |  |
| DBH | 9 | rs1611115 | 136500515 | T | 0,25 | 0,1538 | C | 1,41 | 0,235 | 1,833 | 0,5149 | 0,6682 | 5,03 |  |
| DYX1C1 | 15 | rs57809907 | 55722882 | A | 0,09375 | 0,0641 | C | 0,2957 | 0,5866 | 1,51 | 0,7626 | 0,3388 | 6,733 |  |
| COMT1 | 22 | rs4680 | 19951271 | A | 0,3438 | 0,3718 | G | 0,07712 | 0,7812 | 0,8851 | 0,4398 | 0,3738 | 2,096 |  |
| MAOA | 23 | rs6323 | 43591036 | G | 0,3125 | 0,2564 | T | 0,3599 | 0,5486 | 1,318 | 0,4612 | 0,5338 | 3,255 |  |
| DCDC2 | 6 | rs2274305 | 24291203 | T | 0.3471 | 0.3647 | C | 0.1723 | 0.6781 | 0.9259 | 0.1855 | 0.6436 | 1.332 | **ADHD+Com vs Ctr_ADHD** |
| KIAA0319 | 6 | rs4504469 | 24588884 | T | 0.3155 | 0.3392 | C | 0.3205 | 0.5713 | 0.8978 | 0.1906 | 0.618 | 1.304 |  |
| FOXP2 | 7 | rs12533005 | 114056055 | C | 0.4588 | 0.4137 | G | 1.061 | 0.303 | 1.201 | 0.1783 | 0.8472 | 1.704 |  |
| DBH | 9 | rs1611115 | 136500515 | T | 0.2294 | 0.2194 | C | 0.07435 | 0.7851 | 1.059 | 0.2117 | 0.6996 | 1.604 |  |
| DYX1C1 | 15 | rs57809907 | 55722882 | A | 0.07831 | 0.09921 | C | 0.6399 | 0.4238 | 0.7715 | 0.3251 | 0.408 | 1.459 |  |
| COMT1 | 22 | rs4680 | 19951271 | A | 0.3471 | 0.4157 | G | 2.505 | 0.1135 | 0.7472 | 0.1845 | 0.5205 | 1.073 |  |
| MAOA | 23 | rs6323 | 43591036 | G | 0.3176 | 0.2588 | T | 2.22 | 0.1362 | 1.333 | 0.1933 | 0.9127 | 1.947 |  |
| DCDC2 | 6 | rs2274305 | 24291203 | T | 0,3551 | 0,3647 | C | 0,04361 | 0,8346 | 0,959 | 0,2003 | 0,6477 | 1,42 | **ADHD vs Ctr_ADHD** |
| KIAA0319 | 6 | rs4504469 | 24588884 | T | 0,3456 | 0,3392 | C | 0,01941 | 0,8892 | 1,029 | 0,2031 | 0,6909 | 1,532 |  |
| FOXP2 | 7 | rs12533005 | 114056055 | C | 0,442 | 0,4137 | G | 0,3572 | 0,55 | 1,123 | 0,1936 | 0,7682 | 1,641 |  |
| DBH | 9 | rs1611115 | 136500515 | T | 0,2246 | 0,2194 | C | 0,01752 | 0,8947 | 1,031 | 0,2305 | 0,6562 | 1,62 |  |
| DYX1C1 | 15 | rs57809907 | 55722882 | A | 0,07463 | 0,09921 | C | 0,7506 | 0,3863 | 0,7323 | 0,3609 | 0,3609 | 1,486 |  |
| COMT1 | 22 | rs4680 | 19951271 | A | 0,3478 | 0,4157 | G | 2,082 | 0,1491 | 0,7497 | 0,2 | 0,5065 | 1,11 |  |
| MAOA | 23 | rs6323 | 43591036 | G | 0,3188 | 0,2588 | T | 1,978 | 0,1597 | 1,34 | 0,2088 | 0,8903 | 2,018 |  |

Abbreviations: Chr=chromosome, BP=base pair, A1=allele 1, F_A=Frequency in affected individuals, F_U=Frequency in unaffected individuals, A2=allele 2.OR=Estimated odds ratio for A1, L95=Lower bound of 95% confidence interval for odds ratio, U95= Uper bound of 95% confidence interval for odds ratio, Dys=dyslexia samples, ADHD=Attention Deficit Hyperactivity Disorder samples, Com=Comorbid samples, Ctr__Dys_=dyslexia controls, Ctr__ADHD_=ADHD controls, Ctr__com_=Comorbid controls. Significance values <0.05 are represented in red. Significance trend values<0.1 are represented in bold.
