# Supplementary material for: Genetic association study of dyslexia and ADHD candidate genes in a Spanish cohort: Implications of comorbid samples
Source: PLoS One. 2018 Oct 31;13(10):e0206431. doi: 10.1371/journal.pone.0206431 (PMC6209299; doi:10.1371/journal.pone.0206431)
Supplement: S6 Table — (DOCX) [file pone.0206431.s006.docx]

**S6 Table**. Basic case/control association analysis results for single markers considering only female samples (allelic model).

| **GEN** | CHR | SNP | BP | A1 | F_A | F_U | A2 | CHISQ | **P** | OR | SE | L95 | U95 | **TEST DEFINITION** |
| --- | --- | --- | --- | --- | --- | --- | --- | --- | --- | --- | --- | --- | --- | --- |
| DCDC2 | 6 | rs2274305 | 24291203 | T | 0.3586 | 0.3927 | C | 1.133 | 0.2872 | 0.8646 | 0.1368 | 0.6613 | 1.13 | **Dys+Com vs Ctr_Dys** |
| KIAA0319 | 6 | rs4504469 | 24588884 | T | 0.3021 | 0.3541 | C | 2.75 | 0.09725 | 0.7895 | 0.1427 | 0.5969 | 1.044 |  |
| FOXP2 | 7 | rs12533005 | 114056055 | C | 0.5448 | 0.4849 | G | 3.311 | **0.06882** | 1.271 | 0.1321 | 0.9813 | 1.647 |  |
| DBH | 9 | rs1611115 | 136500515 | T | 0.2138 | 0.2084 | C | 0.04032 | 0.8409 | 1.033 | 0.161 | 0.7533 | 1.416 |  |
| DYX1C1 | 15 | rs57809907 | 55722882 | A | 0.1224 | 0.09571 | C | 1.782 | 0.1819 | 1.318 | 0.2071 | 0.878 | 1.977 |  |
| COMT1 | 22 | rs4680 | 19951271 | A | 0.4586 | 0.4486 | G | 0.09392 | 0.7593 | 1.041 | 0.1322 | 0.8037 | 1.349 |  |
| MAOA | 23 | rs6323 | 43591036 | G | 0.269 | 0.2451 | T | 0.3498 | 0.5542 | 1.133 | 0.2114 | 0.7488 | 1.715 |  |
| DCDC2 | 6 | rs2274305 | 24291203 | T | 0,3664 | 0,3927 | C | 0,5626 | 0,4532 | 0,8941 | 0,1493 | 0,6673 | 1,198 | **Dys vs Ctr_Dys** |
| KIAA0319 | 6 | rs4504469 | 24588884 | T | 0,3261 | 0,3541 | C | 0,6588 | 0,417 | 0,8826 | 0,1539 | 0,6528 | 1,193 |  |
| FOXP2 | 7 | rs12533005 | 114056055 | C | 0,5172 | 0,4849 | G | 0,8039 | 0,3699 | 1,138 | 0,1443 | 0,8577 | 1,51 |  |
| DBH | 9 | rs1611115 | 136500515 | T | 0,2198 | 0,2084 | C | 0,1508 | 0,6977 | 1,07 | 0,1748 | 0,7598 | 1,508 |  |
| DYX1C1 | 15 | rs57809907 | 55722882 | A | 0,114 | 0,09571 | C | 0,7144 | 0,398 | 1,216 | 0,2318 | 0,7721 | 1,916 |  |
| COMT1 | 22 | rs4680 | 19951271 | A | 0,4828 | 0,4486 | G | 0,9066 | 0,341 | 1,147 | 0,1444 | 0,8645 | 1,523 |  |
| MAOA | 23 | rs6323 | 43591036 | G | 0,3017 | 0,2451 | T | 1,623 | 0,2026 | 1,331 | 0,2248 | 0,8566 | 2,067 |  |
| DCDC2 | 6 | rs2274305 | 24291203 | T | 0,3276 | 0,392 | C | 0,9642 | 0,3261 | 0,7555 | 0,2863 | 0,431 | 1,324 | **Com vs Ctr_Dys** |
| KIAA0319 | 6 | rs4504469 | 24588884 | T | 0,2069 | 0,3535 | C | 5,234 | **0,02214** | 0,4772 | 0,3301 | 0,2499 | 0,9113 |  |
| FOXP2 | 7 | rs12533005 | 114056055 | C | 0,6552 | 0,4858 | G | 6,328 | **0,01188** | 2,011 | 0,2826 | 1,156 | 3,499 |  |
| DBH | 9 | rs1611115 | 136500515 | T | 0,1897 | 0,208 | C | 0,1134 | 0,7363 | 0,891 | 0,3429 | 0,4549 | 1,745 |  |
| DYX1C1 | 15 | rs57809907 | 55722882 | A | 0,1552 | 0,09554 | C | 2,209 | 0,1372 | 1,739 | 0,3766 | 0,8312 | 3,638 |  |
| COMT1 | 22 | rs4680 | 19951271 | A | 0,3621 | 0,4487 | G | 1,676 | 0,1955 | 0,6974 | 0,2797 | 0,4031 | 1,207 |  |
| MAOA | 23 | rs6323 | 43591036 | G | 0,1379 | 0,2451 | T | 1,738 | 0,1874 | 0,4928 | 0,5474 | 0,1685 | 1,441 |  |
| DCDC2 | 6 | rs2274305 | 24291203 | T | 0.3276 | 0.3784 | C | 0.5752 | 0.4482 | 0.8004 | 0.2941 | 0.4498 | 1.424 | **Com vs Ctr_ADHD** |
| KIAA0319 | 6 | rs4504469 | 24588884 | T | 0.2069 | 0.3682 | C | 5.949 | **0.01472** | 0.4476 | 0.3368 | 0.2313 | 0.866 |  |
| FOXP2 | 7 | rs12533005 | 114056055 | C | 0.6552 | 0.4614 | G | 7.846 | **0.005093** | 2.218 | 0.29 | 1.256 | 3.915 |  |
| DBH | 9 | rs1611115 | 136500515 | T | 0.1897 | 0.2121 | C | 0.1578 | 0.6911 | 0.8696 | 0.3519 | 0.4363 | 1.733 |  |
| DYX1C1 | 15 | rs57809907 | 55722882 | A | 0.1552 | 0.08333 | C | 3.266 | 0.07072 | 2.02 | 0.3961 | 0.9296 | 4.391 |  |
| COMT1 | 22 | rs4680 | 19951271 | A | 0.3621 | 0.4807 | G | 2.948 | 0.08601 | 0.6132 | 0.287 | 0.3493 | 1.076 |  |
| MAOA | 23 | rs6323 | 43591036 | G | 0.1379 | 0.2519 | T | 1.855 | 0.1731 | 0.4751 | 0.5573 | 0.1594 | 1.416 |  |
| DCDC2 | 6 | rs2274305 | 24291203 | T | 0,3276 | 0,4328 | C | 1,867 | 0,1718 | 0,6384 | 0,3297 | 0,3346 | 1,218 | **Com vs Ctr_Com** |
| KIAA0319 | 6 | rs4504469 | 24588884 | T | 0,2069 | 0,3507 | C | 3,935 | **0,04729** | 0,4829 | 0,3713 | 0,2332 | 0,9997 |  |
| FOXP2 | 7 | rs12533005 | 114056055 | G | 0,3448 | 0,5299 | C | 5,558 | **0,0184** | 0,467 | 0,326 | 0,2465 | 0,8847 |  |
| DBH | 9 | rs1611115 | 136500515 | T | 0,1897 | 0,1439 | C | 0,6334 | 0,4261 | 1,392 | 0,4167 | 0,615 | 3,15 |  |
| DYX1C1 | 15 | rs57809907 | 55722882 | A | 0,1552 | 0,08955 | C | 1,789 | 0,181 | 1,867 | 0,4723 | 0,74 | 4,712 |  |
| COMT1 | 22 | rs4680 | 19951271 | A | 0,3621 | 0,4925 | G | 2,781 | **0,09541** | 0,5848 | 0,3233 | 0,3103 | 1,102 |  |
| MAOA | 23 | rs6323 | 43591036 | G | 0,1379 | 0,2985 | T | 2,783 | **0,09525** | 0,376 | 0,6011 | 0,1158 | 1,221 |  |
| DCDC2 | 6 | rs2274305 | 24291203 | T | 0.3912 | 0.3784 | C | 0.1296 | 0.7189 | 1.055 | 0.15 | 0.7867 | 1.416 | **ADHD+Com vs Ctr_ADHD** |
| KIAA0319 | 6 | rs4504469 | 24588884 | T | 0.3061 | 0.3682 | C | 3.193 | **0.07397** | 0.757 | 0.156 | 0.5575 | 1.028 |  |
| FOXP2 | 7 | rs12533005 | 114056055 | C | 0.4966 | 0.4614 | G | 0.9325 | 0.3342 | 1.152 | 0.1462 | 0.8647 | 1.534 |  |
| DBH | 9 | rs1611115 | 136500515 | T | 0.1633 | 0.2121 | C | 2.845 | **0.09168** | 0.725 | 0.1912 | 0.4984 | 1.055 |  |
| DYX1C1 | 15 | rs57809907 | 55722882 | A | 0.1096 | 0.08333 | C | 1.527 | 0.2166 | 1.354 | 0.2459 | 0.8361 | 2.192 |  |
| COMT1 | 22 | rs4680 | 19951271 | A | 0.4558 | 0.4807 | G | 0.4671 | 0.4943 | 0.9048 | 0.1464 | 0.679 | 1.206 |  |
| MAOA | 23 | rs6323 | 43591036 | G | 0.2449 | 0.2519 | T | 0.02479 | 0.8749 | 0.963 | 0.2395 | 0.6022 | 1.54 |  |
| DCDC2 | 6 | rs2274305 | 24291203 | T | 0,4068 | 0,3784 | C | 0,5511 | 0,4579 | 1,127 | 0,1605 | 0,8224 | 1,543 | **ADHD vs Ctr_ADHD** |
| KIAA0319 | 6 | rs4504469 | 24588884 | T | 0,3305 | 0,3682 | C | 1,004 | 0,3164 | 0,847 | 0,1658 | 0,6121 | 1,172 |  |
| FOXP2 | 7 | rs12533005 | 114056055 | C | 0,4576 | 0,4614 | G | 0,009241 | 0,9234 | 0,985 | 0,1576 | 0,7232 | 1,341 |  |
| DBH | 9 | rs1611115 | 136500515 | T | 0,1568 | 0,2121 | C | 3,153 | **0,07579** | 0,6908 | 0,209 | 0,4586 | 1,041 |  |
| DYX1C1 | 15 | rs57809907 | 55722882 | A | 0,09829 | 0,08333 | C | 0,4488 | 0,5029 | 1,199 | 0,2713 | 0,7046 | 2,041 |  |
| COMT1 | 22 | rs4680 | 19951271 | A | 0,4788 | 0,4807 | G | 0,002299 | 0,9618 | 0,9925 | 0,1572 | 0,7293 | 1,351 |  |
| MAOA | 23 | rs6323 | 43591036 | G | 0,2712 | 0,2519 | T | 0,1567 | 0,6922 | 1,105 | 0,2519 | 0,6744 | 1,81 |  |

Abbreviations: Chr=chromosome, BP=base pair, A1=allele 1, F_A=Frequency in affected individuals, F_U=Frequency in unaffected individuals, A2=allele 2.OR=Estimated odds ratio for A1, L95=Lower bound of 95% confidence interval for odds ratio, U95= Uper bound of 95% confidence interval for odds ratio, Dys=dyslexia samples, ADHD=Attention Deficit Hyperactivity Disorder samples, Com=Comorbid samples, Ctr__Dys_=dyslexia controls, Ctr__ADHD_=ADHD controls, Ctr__com_=Comorbid controls. Significance values <0.05 are represented in red. Significance trend values<0.1 are represented in bold.
