# Supplementary material for: Genetic association study of dyslexia and ADHD candidate genes in a Spanish cohort: Implications of comorbid samples
Source: PLoS One. 2018 Oct 31;13(10):e0206431. doi: 10.1371/journal.pone.0206431 (PMC6209299; doi:10.1371/journal.pone.0206431)
Supplement: S7 Table — (DOCX) [file pone.0206431.s007.docx]

**S7 Table**. Candidate gene´s allele frequencies in European population, based on 1006 samples (source: NCBI).

|  | DCDC2-rs2274305 | | KIAA0319-rs4504469 | | FOXP2-rs12533005 | | DBH-rs1611115 | | DYX1C1-rs57809907 | | COMT-rs4680 | | MAOA-rs6323 | |
| --- | --- | --- | --- | --- | --- | --- | --- | --- | --- | --- | --- | --- | --- | --- |
| **Alleles** | A | C | C | T | C | G | C | T | A | C | A | G | G | T |
| **Frequency** | 0,332 | 0,668 | 0,578 | 0,423 | 0,485 | 0,515 | 0,790 | 0,210 | 0,087 | 0,914 | 0,500 | 0,500 | 0,457 | 0,543 |
