# Supplementary material for: Genetic association study of dyslexia and ADHD candidate genes in a Spanish cohort: Implications of comorbid samples
Source: PLoS One. 2018 Oct 31;13(10):e0206431. doi: 10.1371/journal.pone.0206431 (PMC6209299; doi:10.1371/journal.pone.0206431)
Supplement: S8 Table — (DOCX) [file pone.0206431.s008.docx]

**S8 Table**. Mean values and standard deviation (SD) of the psychometric characteristics across ages for dyslexia and dyslexia-control samples.

|  |  | **DYS** | | | | | | |  |
| --- | --- | --- | --- | --- | --- | --- | --- | --- | --- |
| **psychometric characteristics** | **AGE** | **7-8** | **9** | **10** | **11** | **12** | **13** | **14-16** | **TOTAL** |
|  | **N** | **57** | **49** | **49** | **48** | **40** | **32** | **11** | **286** |
| Efficiency in reading words and pseudowords | Mean | 0.137 | 0.154 | 0.178 | 0.185 | 0.192 | 0.214 | 0.202 |  |
|  | SD | 0.027 | 0.036 | 0.029 | 0.036 | 0.039 | 0.032 | 0.037 |  |
| Rapid naming of pictures and colours (RAN) | Mean | 40583.317 | 37979.474 | 35452.566 | 34184.876 | 31346.439 | 29885.85 | 31145.082 |  |
|  | SD | 5284.269 | 4728.604 | 4194.977 | 5353.819 | 4873.097 | 5035.016 | 5988.608 |  |
| Reaction time in phoneme picture matching for phonological awareness (PA) | Mean | 2900.609 | 2641.186 | 2460.775 | 2303.334 | 2220.365 | 2217.49 | 2131.743 |  |
|  | SD | 542.707 | 563.441 | 482.488 | 489.129 | 508.428 | 575.332 | 509.572 |  |
| Accuracy in letter position identification | Mean | -26.433 | -21.361 | -25.986 | -27.083 | -19.833 | -23.333 | -19.394 |  |
|  | SD | 24.364 | 21.212 | 22.618 | 18.726 | 20.17 | 16.417 | 9.167 |  |
| Reaction time in syllable identification | Mean | 1140.861 | 1170.399 | 1090.605 | 1051.714 | 989.522 | 947.671 | 999.952 |  |
|  | SD | 186.181 | 233.509 | 220.626 | 195.277 | 214.32 | 235.293 | 195.651 |  |
|  |  |  |  |  |  |  |  |  |  |
|  |  | **DYS-CONTROL** | | | | | | |  |
| **psychometric characteristics** | **AGE** | **7-8** | **9** | **10** | **11** | **12** | **13** | **14-16** | **TOTAL** |
|  | **N** | **190** | **218** | **208** | **210** | **199** | **168** | **44** | **1237** |
| Efficiency in reading words and pseudowords | Mean | 0.238 | 0.261 | 0.278 | 0.297 | 0.316 | 0.326 | 0.31 |  |
|  | SD | 0.039 | 0.037 | 0.036 | 0.037 | 0.035 | 0.033 | 0.034 |  |
| Rapid naming of pictures and colours (RAN) | Mean | 31691.745 | 29614.89 | 28181.451 | 25960.613 | 24188.126 | 22826.411 | 23390.544 |  |
|  | SD | 4752.097 | 4214.306 | 4144.138 | 3759.098 | 3688.679 | 3115.825 | 4528.629 |  |
| Reaction time in phoneme picture matching for phonological awareness (PA) | Mean | 2272.575 | 2136.107 | 1995.088 | 1790.608 | 1658.728 | 1483.531 | 1566.11 |  |
|  | SD | 485.74 | 514.913 | 451.651 | 448.399 | 410.964 | 338.546 | 365.566 |  |
| Accuracy in letter position identification | Mean | -27.439 | -22.385 | -18.397 | -16.825 | -17.688 | -14.921 | -18.485 |  |
|  | SD | 20.832 | 19.789 | 15.326 | 15.408 | 15.243 | 13.298 | 16.13 |  |
| Reaction time in syllable identification | Mean | 1135.128 | 1086.692 | 1031.825 | 970.065 | 914.729 | 857.836 | 875.132 |  |
|  | SD | 262.297 | 237.712 | 190.108 | 180.311 | 173.413 | 147.35 | 231.254 |  |
