# Supplementary material for: Genetic association study of dyslexia and ADHD candidate genes in a Spanish cohort: Implications of comorbid samples
Source: PLoS One. 2018 Oct 31;13(10):e0206431. doi: 10.1371/journal.pone.0206431 (PMC6209299; doi:10.1371/journal.pone.0206431)
Supplement: S9 Table — (DOCX) [file pone.0206431.s009.docx]

**S9 Table**. Mean values and standard deviation (SD) of the psychometric characteristics across ages for ADHD and ADHD-control samples.

|  |  | **ADHD** | | | | | | |  |
| --- | --- | --- | --- | --- | --- | --- | --- | --- | --- |
| **psychometric characteristics** | **AGE** | **7-8** | **9** | **10** | **11** | **12** | **13** | **14-16** | **TOTAL** |
|  | **N** | **72** | **22** | **24** | **25** | **23** | **21** | **1** | **188** |
| Efficiency in reading words and pseudowords | Mean | 0.172 | 0.206 | 0.22 | 0.248 | 0.255 | 0.276 | 0.346 |  |
|  | SD | 0.042 | 0.033 | 0.051 | 0.034 | 0.033 | 0.035 |  |  |
| Rapid naming of pictures and colours (RAN) | Mean | 38065.47 | 35128.461 | 33481.443 | 30224.896 | 29005.39 | 24159.016 | 30806.9 |  |
|  | SD | 5083.809 | 4893.722 | 3524.726 | 5407.665 | 3964.519 | 3015.467 |  |  |
| Reaction time in phoneme picture matching for phonological awareness (PA) | Mean | 2768.474 | 2749.921 | 2385.898 | 2219.54 | 2162.312 | 1861.202 | 1556.878 |  |
|  | SD | 688.211 | 683.914 | 414.651 | 517.031 | 559.11 | 435.784 |  |  |
| Accuracy in letter position identification | Mean | -29.84 | -30 | -26.944 | -14.667 | -16.522 | -15.789 | 0 |  |
|  | SD | 26.73 | 20 | 18.176 | 13.333 | 15.025 | 16.362 |  |  |
| Reaction time in syllable identification | Mean | 1342.184 | 1351.392 | 1183.872 | 1166.418 | 1069.427 | 918.767 | 855.52 |  |
|  | SD | 267.506 | 242.782 | 372.432 | 358.233 | 244.367 | 142.992 |  |  |
|  |  |  |  |  |  |  |  |  |  |
|  |  | **ADHD-CONTROL** | | | | | | |  |
| **psychometric characteristics** | **AGE** | **7-8** | **9** | **10** | **11** | **12** | **13** | **14-16** | **TOTAL** |
|  | **N** | **265** | **62** | **69** | **51** | **37** | **34** | **7** | **525** |
| Efficiency in reading words and pseudowords | Mean | 0.187 | 0.216 | 0.238 | 0.235 | 0.256 | 0.282 | 0.322 |  |
|  | SD | 0.049 | 0.049 | 0.05 | 0.031 | 0.046 | 0.043 | 0.042 |  |
| Rapid naming of pictures and colours (RAN) | Mean | 35974.537 | 32327.26 | 29836.33 | 27135.068 | 27219.39 | 24363.374 | 21713.03 |  |
|  | SD | 5750.78 | 5344.464 | 5044.271 | 3922.733 | 4970.65 | 3322.119 | 4685.078 |  |
| Reaction time in phoneme picture matching for phonological awareness (PA) | Mean | 2640.526 | 2325.667 | 2111.201 | 1806.41 | 1819.349 | 1536.589 | 1560.422 |  |
|  | SD | 613.816 | 563.289 | 468.63 | 469.015 | 483.049 | 334.846 | 705.232 |  |
| Accuracy in letter position identification | Mean | -28.973 | -25.914 | -23.795 | -21.25 | -18.739 | -16.989 | -29.333 |  |
|  | SD | 21.22 | 22.875 | 20.275 | 23.243 | 21.305 | 13.967 | 11.155 |  |
| Reaction time in syllable identification | Mean | 1146.432 | 1024.372 | 979.739 | 927.297 | 859.805 | 856.686 | 748.228 |  |
|  | SD | 262.402 | 188.296 | 180.2 | 189.165 | 135.851 | 109.788 | 99.027 |  |
