# Supplementary material for: Genetic association study of dyslexia and ADHD candidate genes in a Spanish cohort: Implications of comorbid samples
Source: PLoS One. 2018 Oct 31;13(10):e0206431. doi: 10.1371/journal.pone.0206431 (PMC6209299; doi:10.1371/journal.pone.0206431)
Supplement: S10 Table — (DOCX) [file pone.0206431.s010.docx]

**S10 Table**. Mean values and standard deviation (SD) of the psychometric characteristics across ages for comorbid and comorbid-control samples.

|  |  | **COMORBID** | | | | | | |  |
| --- | --- | --- | --- | --- | --- | --- | --- | --- | --- |
| **psychometric characteristics** | **AGE** | **7-8** | **9** | **10** | **11** | **12** | **13** | **14-16** | **TOTAL** |
|  | **N** | **10** | **8** | **7** | **9** | **4** | **6** | **1** | **45** |
| Efficiency in reading words and pseudowords | Mean | 0.135 | 0.155 | 0.177 | 0.181 | 0.194 | 0.214 | 0.251 |  |
|  | SD | 0.021 | 0.026 | 0.013 | 0.019 | 0.049 | 0.035 |  |  |
| Rapid naming of pictures and colours (RAN) | Mean | 43216.745 | 37834.475 | 36437.979 | 36550.806 | 34640.213 | 27498.267 | 35071.65 |  |
|  | SD | 4278.878 | 5660.372 | 4135.046 | 6123.186 | 4569.59 | 3029.259 |  |  |
| Reaction time in phoneme picture matching for phonological awareness (PA) | Mean | 3063.483 | 2754.906 | 2423.788 | 2409.207 | 2171.042 | 1966.527 | 2335.551 |  |
|  | SD | 406.196 | 490.291 | 641.219 | 390.054 | 666.554 | 294.716 |  |  |
| Accuracy in letter position identification | Mean | -32 | -22.5 | -29.524 | -25.185 | -21.667 | -17.778 | -13.333 |  |
|  | SD | 22.399 | 17.067 | 29.023 | 20.488 | 11.386 | 14.402 |  |  |
| Reaction time in syllable identification | Mean | 1213.832 | 1192.474 | 1090.713 | 1120.103 | 1096.689 | 910.658 | 1114.097 |  |
|  | SD | 206.01 | 177.938 | 103.745 | 236.523 | 320.927 | 112.452 |  |  |
|  |  |  |  |  |  |  |  |  |  |
|  |  | **COMORBID-CONTROL** | | | | | | |  |
| **psychometric characteristics** | **AGE** | **7-8** | **9** | **10** | **11** | **12** | **13** | **14-16** | **TOTAL** |
|  | **N** | **20** | **21** | **14** | **21** | **20** | **16** | **1** | **113** |
| Efficiency in reading words and pseudowords | Mean | 0.19 | 0.223 | 0.252 | 0.246 | 0.265 | 0.282 | 0.335 |  |
|  | SD | 0.037 | 0.035 | 0.042 | 0.023 | 0.021 | 0.027 |  |  |
| Rapid naming of pictures and colours (RAN) | Mean | 33568.637 | 32538.752 | 29076.268 | 27534.295 | 26701.938 | 25549.369 | 18801.55 |  |
|  | SD | 3327.303 | 6073.652 | 4666.747 | 4315.237 | 3943.915 | 3014.214 |  |  |
| Reaction time in phoneme picture matching for phonological awareness (PA) | Mean | 2555.092 | 2307.949 | 2198.277 | 1832.767 | 1719.152 | 1581.063 | 1626.113 |  |
|  | SD | 594.775 | 486.235 | 441.355 | 391.849 | 347.287 | 330.859 |  |  |
| Accuracy in letter position identification | Mean | -30.333 | -28.571 | -27.619 | -17.778 | -15.333 | -13.333 | -33.333 |  |
|  | SD | 19.524 | 18.275 | 20.399 | 24.525 | 23.998 | 12.172 |  |  |
| Reaction time in syllable identification | Mean | 1107.72 | 1011.636 | 941.997 | 940.084 | 854.652 | 836.473 | 804.398 |  |
|  | SD | 323.912 | 169.468 | 150.722 | 109.489 | 165.154 | 110.25 |  |  |
