# Supplementary material for: Genetic association study of dyslexia and ADHD candidate genes in a Spanish cohort: Implications of comorbid samples
Source: PLoS One. 2018 Oct 31;13(10):e0206431. doi: 10.1371/journal.pone.0206431 (PMC6209299; doi:10.1371/journal.pone.0206431)
Supplement: S1 File — (DOCX) [file pone.0206431.s011.docx]

**S1 Supporting Information. Description of cognitive tasks**

1. **Word and Pseudoword Reading:** Participants had to read a list of 192 strings of letters that appeared in the middle of the screen, some of which were real words (96) and some of which were pseudowords (96). Pseudowords were pronounceable combinations of letters that did not represent Spanish words but that could be articulated using the pronunciation rules of Spanish (i.e., all the pseudowords were orthotactically and phonotactically valid). Furthermore, half of the words were of high frequency (e.g., “nariz”, “bolsa”, “prisa” and “dedo”), and half of the words were or low frequency (e.g., “tazón”, “gamba”, “gramo” and “puño”). Several lexical and sub-lexical factors were carefully controlled for in each of the subsets of items. Word and pseudoword reading was included to assess specifically reading and the grapheme/phoneme decoding processes. In each trial, a centered fixation cross was first displayed for 500ms. Immediately after that, the letter string was presented in lowercase Courier New font until a response was given or for a maximum of 3000ms. Verbal responses were recorded for posterior analysis of naming latencies and accuracy rates. The order of string presentation was randomized across participants. Prior to the experimental items, participants received words and pseudowords as practice trials.
2. **Rapid automatized naming (RAN) of Pictures and Colors:** Children had to name as fast as possible from left to right and from top to bottom the colors or drawings that appeared in four arrays, each with one of these kinds of stimuli. Each array consisted of 6 different elements, repeatedly presented in a 9-column x 4-row matrix (leading to a total of 36 elements to be named). Each element was presented 6 times in each array. The colors used were red, brown, green, blue, yellow and black. The pictures displayed were a tree (“árbol”), a finger (“dedo”), a watch (“reloj”), a button (“botón”), a house (“casa”) and a glass (“vaso”). Before the task was administered, a practice session was run so that the experimenters made sure that participants knew the correct names of the colors and pictures that were going to be displayed during the experiment. Verbal responses were recorded for posterior analysis of naming latencies and accuracy rates. Participants had a maximum of 1 minute to name the elements of each array. The order of array presentation was randomized across participants. Naming time was coded from the onset of the first utterance to the offset of the last one.
3. **Phonological Awareness (Phoneme Picture Matching):** Two pictures were presented simultaneously on the screen and a word was presented auditorily. Participants had to choose the picture whose name started with the same initial phoneme than the initial phoneme of the word presented auditorily. Twenty different trials were created, each of them associated with a previously recorded word. The auditorily presented words (e.g., “pala”, translated as “shovel”) were common words that always started with a CV sequence. The names of the visually presented pictures also started with a CV sequence (e.g., the pictures of a “pig” and a “pineapple”: “cerdo” and “piña”, respectively). In all trials, the first phoneme of the word that was presented auditorily matched the first phoneme of the name of one of the pictures displayed (e.g., “pala” and “piña”). Each of the pictures was presented on one side of the screen, and participants had to press a button in the keyboard corresponding to the location of the correct picture (either right or left button). The number of expected right and left responses was controlled for in order to avoid any response bias. In each trial, participants first saw a fixation cross that stayed in the center of the screen for 250ms. Next, the auditory word was presented time-locked to the presentation of the two pictures on the left and right sides of the screen. The next trial started after 5000ms or once a response was given. Prior to the experiment, participants completed a practice with different trials.
4. **Syllable Discrimination:** Participants had to decide if two sequentially auditorily presented syllables were the same or not (e.g., “pe”-“pe” vs. “pe”-“be”). This is a task that evaluates the listeners’ ability to discriminate consonant contrasts in the context of pairs of syllables. 26 trials were administered to each participant (13 requiring a “same” response, and 13 requiring a “different” response. The syllables that were used for the “same” trials were: “pe”, “po”, “pu”, “nu”, “bu”, “do”, “sa”, “mu”, “fa”, “ma”, “to”, “na” and “fo”. These same syllables were also used as referents for the “different” trials, paired with the following mismatching targets: “be”, “bo”, “bu”, “mu”, “pu”, “to”, “fa”, “nu”, “sa”, “na”, “do”, “ma” and “so”. As can be seen, only the initial phoneme of each of the syllables differed in the “different” trials. Furthermore, the similarity between the mismatching syllables was kept constant so that the initial phonemes shared the place of articulation. In each trial, participants first saw a fixation cross that lasted in the center of the screen for 500ms. Next, the first auditory syllable was presented. A silence of 500ms was left at the offset of the first syllable and immediately after it the second syllable was presented. The next trial started after 4000ms or once a response was given. Participants had to press one out of two keyboard buttons in order to indicate whether the two syllables were identical or different. Prior to the experiment, participants completed a practice with different trials.
5. **Verbal Stroop:** Participants had to name as fast as possible the colors of stimuli presented on screen. Participants were visually presented with printed strings that could refer to the name of a color (the words “azul”, “amarillo”, “verde” and “rojo”, translated as “blue”, “yellow”, “green” and “red”, respectively) or to other concepts (the words “sala”, “torno”, “olor” and “uniforme”, translated as “living room”, “lathe”, “smell” and “uniform”, respectively). The two sets of words (namely, color names and concept names) were matched for length and word frequency. Additionally, some of the stimuli were a simple line of percentage symbols (i.e., “%%%%%%”). The words corresponding to the colors could be presented with an ink color that was either congruent or incongruent in relation to the written concept. The words corresponding to other concepts were also presented in different ink colors, and were used as a neutral condition since no congruency manipulation was carried out for them. Similarly, the strings of percentage symbols were also presented in different ink colors, and were used as another baseline. A total of 96 trials were administered to the participants, 24 corresponded to a congruent condition (i.e., match between the color name and the ink color), 24 to an incongruent condition (i.e., mismatch between the color name and the ink color), 24 to a neutral word condition, and 24 to a neutral symbol string condition. In each trial, participants first saw a fixation cross that lasted in the center of the screen for 250ms. Next, the critical item was displayed for a maximum of 2500ms. They were instructed to say the color of the ink as fast as possible, and verbal responses were recorded for posterior analysis of naming latencies and accuracy rates. Prior to the experiment, participants completed a practice with different trials.
6. **Numerical Stroop:** Participants judged the physical size of two numbers presented on screen side-by-side by indicating which was largest in size (i.e., which of the two numbers was physically bigger than the other). Materials included all the single-digit numbers except for 0 and 5. The number pairs could include the same number (neutral condition; e.g., 3 and 3), or different numbers (congruent and incongruent conditions). A total of 48 trials were displayed to each participant (16 neutral, 16 congruent and 16 incongruent). The location of the correct digit was controlled for, so that in half of the trials the biggest number was displayed on the left side of the screen and in the other half it was presented on the right. In the congruent trials the physically biggest number was also the number referring to the highest quantity (i.e., largest in size is the largest in quantity; e.g., 1 and 6). In contrast, in the incongruent trials the physically largest number was not the one referring to the highest value or magnitude (e.g., 1 and 6). Number pairs in the congruent and incongruent trials numerically differed at least in two numbers according to the number line. In each trial, participants first saw a fixation cross that stayed in the center of the screen for 250ms. Next, the critical item was displayed for a maximum of 2500ms. They were instructed to indicate as fast as possible which of the two digits was the physically biggest one by pressing one out of two buttons in the keyboard. Prior to the experiment, participants completed a practice with different trials.
7. **ANT (Attentional Network Task)**: Each trial began with a central cross (1° of visual angle). The cue was an asterisk (1°) that could be presented at the same position of the upcoming target (valid condition), in the opposite position (invalid condition), above and below the fixation cross (double cue condition), at the center of the screen (neutral conditions), or it could be absent (no-cue condition). The target was a left or right pointing yellow fish (1.6°), presented above or below fixation. The central fish was presented flanked on both sides by two fish pointing either in the same (congruent trials), or in the opposite direction (incongruent trials). The distance between the fish was 0.21°. The target and flankers subtended 8.84° and were presented 1° above or below the fixation cross over a blue-green background. Each trial began with a fixation period of random variable duration (400-1600 ms). This was followed by a warning cue presented for 150 ms. After a fixation period of 450 ms, the target and flankers were displayed until response, to a maximum of 1700 ms. After responding, participants received positive or negative visual feedback from the computer. A session of the ANT consisted of a total 24 practice trials and three experimental blocks of 48 trials each. Each trial represented one of 10 conditions in equal proportions: two flanker conditions (congruent, incongruent) x five cue conditions (no-cue, central cue, double cue, valid cue, and invalid cue). The practice block took approximately 3 min and each experimental block took approximately 5 min. Participants viewed the screen from a distance of about 53 cm and indicated their responses via a right (“L”) or left (“S”) button of the keyboard, depending on the direction of the central fish. Accuracy and reaction time were recorded.
