# Supplementary figures and images for: Genetic association study of dyslexia and ADHD candidate genes in a Spanish cohort: Implications of comorbid samples
Source: PLoS One. 2018 Oct 31;13(10):e0206431. doi: 10.1371/journal.pone.0206431 (PMC6209299; doi:10.1371/journal.pone.0206431)

# Principal Component Analysis

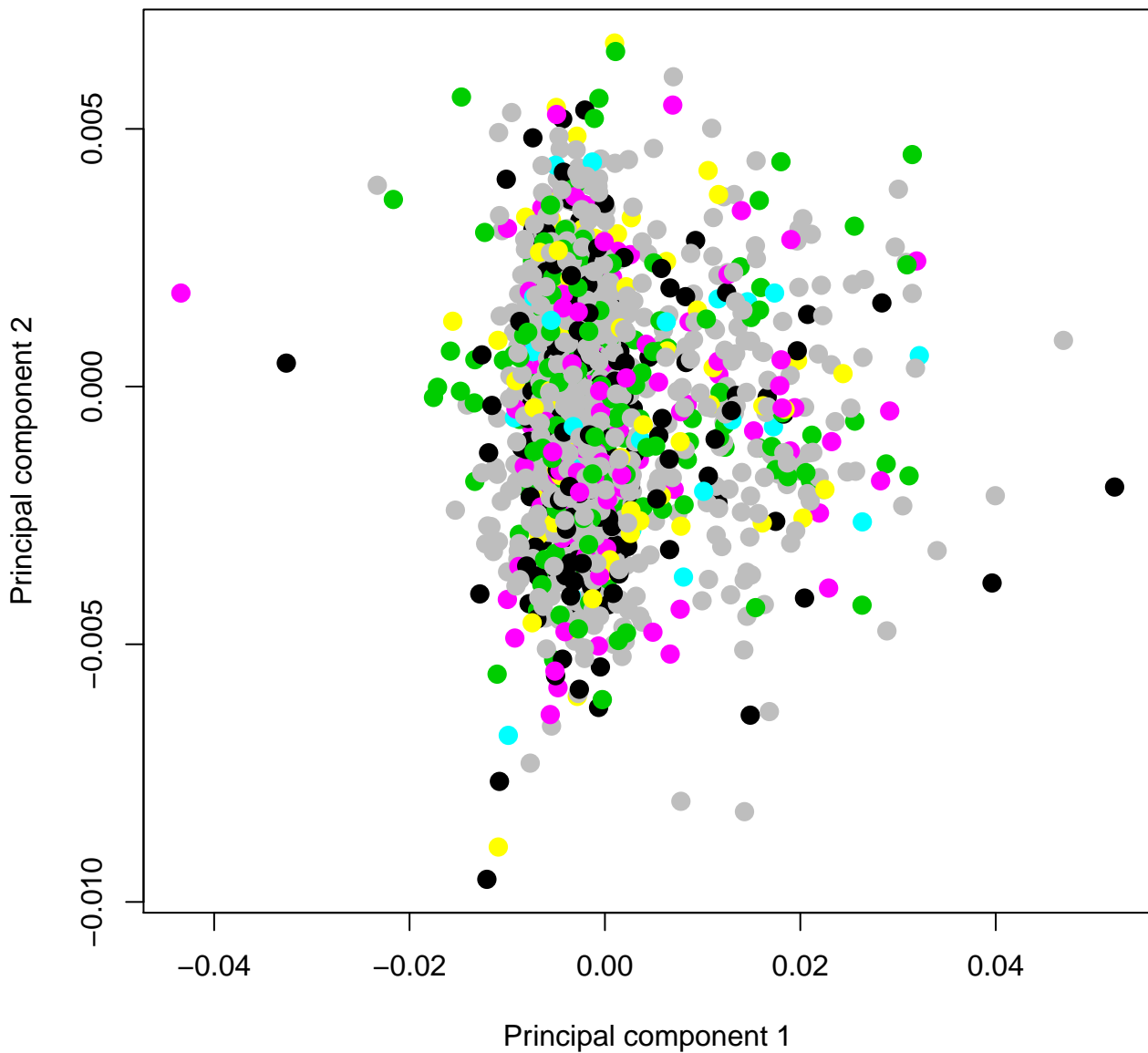

Supplement: S1 Fig — Graphic representation of the two principal components obtained through the PLINK program for the cases and controls of dyslexia, ADHD and comorbidity after whole genome genotyping with OmniExpress Beadchips (Illumina Inc.). Green dots = Dyslexia; grey = Dyslexia controls; pink = ADHD; black = ADHD controls; blue = comorbids; yellow = Comorbid controls. (PDF) [file pone.0206431.s012.pdf]
